# ASD-like behaviors, a dysregulated inflammatory response and decreased expression of PLP1 characterize mice deficient for sialyltransferase ST3GAL5

**DOI:** 10.1016/j.bbih.2021.100306

**Published:** 2021-07-27

**Authors:** Tatyana Strekalova, Evgeniy Svirin, Ekaterina Veniaminova, Ekaterina Kopeikina, Tatyana Veremeyko, Amanda W.Y. Yung, Andrey Proshin, Susanne Walitza, Daniel C. Anthony, Lee Wei Lim, Klaus-Peter Lesch, Eugene D. Ponomarev

**Affiliations:** a Department of Psychiatry and Neuropsychology, School for Mental Health and Neuroscience (MHeNS), Maastricht University, Maastricht, the Netherlands; b Laboratory of Psychiatric Neurobiology, Institute of Molecular Medicine and Department of Normal Physiology, Sechenov First Moscow State Medical University, Moscow, Russia; c Division of Molecular Psychiatry, Center of Mental Health, University of Würzburg, Würzburg, Germany; d School of Biomedical Sciences, Faculty of Medicine, The Chinese University of Hong Kong, Shatin, Hong Kong; e P.K. Anokhin Research Institute of Normal Physiology, Moscow, Russia; f Department for Child and Adolescent Psychiatry and Psychotherapy of the University of Zurich and the University Hospital of Psychiatry, Zurich, Switzerland; g Department of Pharmacology, Oxford University, Oxford, United Kingdom; h Neuromodulation Laboratory, School of Biomedical Sciences, Li Ka Shing Faculty of Medicine, The University of Hong Kong, Hong Kong; i Kunmin Institute of Zoology, Chinese University of Hong Kong Joint Laboratory of Bioresources and Molecular Research of Common Diseases, Kunmin-Hong Kong, China

**Keywords:** Lactosylceramide alpha-2,3-sialyltransferase (ST3GAL5), Major brain gangliosides, Autism spectrum disorder (ASD), Aggression, Proteolipid protein 1 (Plp1), Neuroinflammation, Mice

## Abstract

Gangliosides are glycosphingolipids, which are abundant in brain, are known to modulate ion channels and cell-to-cell communication. Deficiencies can result in aberrant myelination and altered immune responses, which can give rise to neurodevelopmental psychiatric disorders. However, to date, little mechanistic data is available on how ganglioside deficiencies contribute to the behavioural disorders. In humans, the loss of lactosylceramide-alpha-2,3-sialyltransferase (ST3Gal5) leads to a severe neuropathology, but in ST3Gal5 knock-out (*St3gal5−/−*) mice the absence of GM3 and associated a-, b- and c-series gangliosides is partially compensated by 0-series gangliosides and there is no overt behavioural phenotype. Here, we sought to examine the behavioural and molecular consequences of GM3 loss more closely. Mutants of both sexes exhibited impaired conditioned taste aversion in an inhibitory learning task and anxiety-like behaviours in the open field, moderate motor deficits, abnormal social interactions, excessive grooming and rearing behaviours. Taken together, the aberrant behaviours are suggestive of an autism spectrum disorder (ASD)-like syndrome. Molecular analysis showed decreased gene and protein expression of proteolipid protein-1 (*Plp1*) and over expression of proinflammatory cytokines, which has been associated with ASD-like syndromes. The inflammatory and behavioural responses to lipopolysaccharide (LPS) were also altered in the *St3gal5−/−* mice compared to wild-type, which is indicative of the importance of GM3 gangliosides in regulating immune responses. Together, the *St3gal5−/−* mice display ASD-like behavioural features, altered response to systemic inflammation, signs of hypomyelination and neuroinflammation, which suggests that deficiency in a- and b-series gangliosides could contribute to the development of an ASD-like pathology in humans.

## Highlights

• *St3gal5*−/− mice exhibit aberrant social, motor and cognitive behavior that is reminiscent of ASD-like syndrome.
• Interleukin1β is upregulated in the brain and spleen of *St3gal5−/−* of both sexes.
• Mutants display reduced gene and protein expression of the myelin protein Plp1.
• LPS induces sex-dependent abnormalities in the inflammatory response and social behavior in the *St3gal5−/−* mice.
• *St3gal5−/−* mice can be used to study the behavioural consequence of a- and b-series ganglioside deficiency

## 1 Introduction

Gangliosides are glycosphingolipids that facilitate neuronal membrane protein organization, signalling and adhesion (Lopez and Báez, 2018; Schnaar et al., 2014). Brain gangliosides also regulate microglia- and cytokine-mediated immune responses, including microglial activation, myelination, CNS development, and platelet activation (Bowser et al., 2019; Dukhinova et al., 2018; Kim et al., 2002). The ganglioside GM3, is generated by alpha-2,3-sialyltransferase 5 (ST3GAL5) or GM3-synthase (Ishii et al., 1999), which is the precursor of the principal brain gangliosides including GM1, GD1a, GD1b, GD3, GT1b and GQ1b. Abnormalities in the *ST3GAL5* gene disrupt GM3 synthesis, leading to intellectual disability, microcephaly, epilepsy, blindness and deafness, somatic growth failure and metabolic syndrome (Gordon-Lipkin et al., 2018; Trinchera et al., 2018).

Emerging evidence suggests that ganglioside deficiency may have other effects in addition to severe neuropathologies observed in individuals with *ST3GAL5* deficiency. Recent GWAS studies have reported an association between SNPs encoding alpha-2,3-sialyltransferase 3 and factors regulating GM function, and the incidence of schizophrenia, attention-deficit/hyperactivity disorder (ADHD) or autism spectrum disorders (ASD). In particular, in a large-scale integrative analysis of genome-wide association studies (GWAS), comprising of 20,183 ADHD cases and 35,191 controls, using DEPICT analysis of gene prioritization, pathway and tissue/cell type enrichment analysis, ST3GAL3 was the top gene associated wirth ADHD (P ​= ​1.19 ​× ​10^−2^) (Zhao et al., 2018). Furthermore, the homozygous loss-of-function mutation of SLC39A8, a well-established schizophrenia biomarker, was shown to result in serum manganese (Mn) abnormalities, a causal factor of glycosyltransferases dysfunction. This mechanism was suggested to underlie the pathophysiology of schizophrenia (Mealer et al., 2020). A more recent GWAS also revealed a relationship between increased expression of a ST3GAL3 transcript in the human fetal brain and a risk for ADHD and schizophrenia (Hall et al., 2020). Moreover, children with ASD often displayed increased anti-ganglioside antibody levels (Mostafa and Al-Ayadhi, 2011; Yang et al., 2018) and changes in the ganglioside expression has also been proposed as a biomarker for schizophrenia (Sarbu et al., 2018).

The mechanisms underlying a link between the path physiology of neurodevelopmental disorders and dysregulated brain gangliosides implicate neuroinflammation as a risk factor of these pathologies (Dunn et al., 2019; Matta et al., 2019; Meyer, 2013), as gangliosides are known to regulate immune responses in which they have an anti-inflammatory role (Galleguillos et al., 2020; Sipione et al., 2020). Specifically, gangliosides have been shown to regulate the effects of tumour necrosis factor (TNF) (Tagami et al., 2002), microglial and platelet activation (Dukhinova et al., 2018; Sotnikov et al., 2013), nitric oxide synthase (NOS2), and ICAM-1 and MCP-1-mediated signalling (Kim et al., 2002; Ryu et al., 2002). For microglia treated with lipopolysaccharide (LPS), interleukin-1β (IL-1β) or ATP, gangliosides have been shown to have anti-inflammatory effects (Galleguillos et al., 2020). Moreover, neuroinflammation and neuro-immune system dysregulation are particularly well established as clinical features of ASD (Gevezova et al., 2020; Siniscalco et al., 2018). However, mechanistic studies of the role of the principal CNS gangliosides in neurodevelopmental psychiatric syndromes are still lacking.

ST3GAL5 dysfunction in humans has been modelled in *St3gal5−/−* mice (Yamashita et al., 2003), but these mutants only partly re-capitulate clinical abnormalities, which may be attributable to the compensatory synthesis of 0-series gangliosides GD1α and GM1b. However, *St3gal5−/−*mice do lack the major CNS gangliosides GM3, GM1, GD1a, GD3, GT1b, GQ1b and display alterations in activity, insulin receptor sensitivity, platelet activation and neuronal damage following brain trauma (Dukhinova et al., 2018; Kopeikina et al., 2020; Niimi et al., 2011; Trinchera et al., 2018). While *St3gal5−/−* mice appear to only to display selective neurodegeneration in the Organ of Corti, they do afford the opportunity to explore the consequences of the brain ganglioside deficiency rather than complete absence (Niimi et al., 2011).

Here, we hypothesized that *St3gal5−/−* mice would display features of neuropsychiatric developmental pathologies and that these changes, given the anti-inflammatory actions of brain gangliosides, would be exacerbated by an inflammatory challenge with LPS. Therefore, we studied male and female *St3gal5−/−* mice for social, restricted-repetitive behaviours to determine whether the animals would display ASD-like features (Haratizadeh et al., 2021), as well as evaluation of inhibitory learning and the expression of myelination and inflammation markers in brain cortex and spleen.

## 2 Methods

### 2.1 Animals

Eight-twelve-week-old *St3gal5−/−* and C57BL/6 mice were bred as described elsewhere (Dukhinova et al., 2018); six-week-old C57BL/6 mice were used in social interaction test. Mice were housed under standard conditions; all protocols complied 2010/63/EU and ARRIVE guidelines (*see* Supplementary file, SF).

### 2.2 Study design

Mice were examined for social interaction, rearing and conditioned taste aversion (Fig. S1A); in the separate groups, open field grooming, motor performance, the mRNA levels of myelination factors and protein concentrations of Plp1 in the brain cortex were studied. A cohort of mice was investigated for social behavior or mRNA expression of pro-inflammatory cytokines in the cortex and spleen 6h following the i.p. administration of LPS (0.1 ​mg/kg) or PBS (Fig. S1B). For protocols used and group sizes, see SF and Figure legends; on average, 9 mice per group were used for the behavioural studies and 5 mice per group were used in molecular assays.

### 2.3 Behavioural tests

Experiments were performed during the dark period of light cycle, followed by offline analysis (Anymaze, Stoelting, Dublin, Ireland); customized equipment was used. Briefly, in the social interaction test, unfamiliar naïve juvenile mouse of the same sex was exposed to adult mice after its habituation to the apparatus, for 10 ​min (Couch et al., 2016; Veniaminova et al., 2017). The number of rears, latency, total duration, and number of episodes of attacking behavior and dominant-like behaviours: following and mounting, and neutral social exploration: nose-nose and nose-anal contacts were recorded. The number of mice displaying following and mounting were scored in the study with LPS. Inhibitory learning was studied in the conditioned taste aversion model, where mice were injected with LiCl, causing nausea, after their exposure to a sucrose solution. A loss of sucrose preference thereafter was taken as a memory measure. Grooming scores in the open field center and time spent therein were investigated. To assess motor functions, latencies to fall or to descend and the incidence of falling or sliding were studied in the Pole and Wire tests, respectively (de Munter et al., 2020, Veniaminova et al., 2020).

### 2.4 Induction of systemic inflammation

Mice were injected with LPS dissolved in PBS (Sigma, St.Louis, MO, USA) at the dose 0.1 ​mg/kg, i.p., or PBS (Couch et al, 2013, 2016). Previous studies reported a marked pro-inflammatory response both in the brain and peripheral tissue 6 ​h after the administration of LPS (Couch et al., 2013). At this time point, significant changes in floating behavior, novelty exploration and social interaction were observed in the mice (Couch et al., 2013; Strekalova and Anthony, *unpublished results*).

### 2.5 Real-time polymerase chain reaction (RT-PCR) and western blot

Mice were terminally anaesthetized with isoflurane inhalation and perfused intracardially with 20 ​ml of PBS, brain cortex and spleen were dissected, homogenized, and stored until use under -80C° (Dukhinova et al., 2018; for details on brain cortex disection, see SF, Fig. S2). RT-PCR of samples of brain cortex and spleen were examined for the expression of IL-1β, IL-6 and TNF and Western blot analysis of Plp1 expression in the brain cortex were performed (Ponomarev et al., 2011; Veremeyko et al., 2018). The pro-inflammatory cytokines were selected for gene expression analysis based on their pivotal roles in local and systemic immune processes (Kany et al., 2019) and for their established association with neuropsychiatric disorders (Dunn et al., 2019; Matta et al., 2019). Furthermore, previous studies in our laboratories have revealed the presence of altered gene expression of IL-1β, IL-6 and TNF in mice with exhibiting aberrant social, emotional and cognitive features following exposure to stress, LPS challenge, or other adverse conditions (Couch et al, 2013, 2016; Gorlova et al., 2019; Pavlov et al, 2017, 2019). Relative gene expression was calculated using the ΔΔC_T_ method and normalized to the expression of glyceraldehyde 3-phosphate dehydrogenase (GAPDH) housekeeping gene and to the expression of the control sample, see Table S1. The choice of the reference gene was based on our previous experiments, in which, in comparison with other houskeeping genes, GAPDH demonstrated relatively stable expression in the tissue obtained in mouse models of systemic inflammation and stress (Couch et al, 2013, 2016; Pavlov et al., 2017). Relative expression of Plp1 protein was calculated as fold change relative to β-actin.

### 2.6 Statistics

Data were analyzed using GraphPad Prism v.8.01 (San Diego, CA, USA). Two- or three-way ANOVA was employed for multiple group comparisons, followed by Tukey's or Sidak's tests, respectively. Data were checked for normality using Shapiro-Wilk test. Data from LPS-treated groups were additionally normalized to PBS-treated groups of the respective genotypes. The level of significance was p ​< ​0.05.

## 3 Results

### 3.1 St3gal5−/− mice display anxiety-like behavior, stereotypy and abnormalities in learning and social interaction

**Fig. 1:**
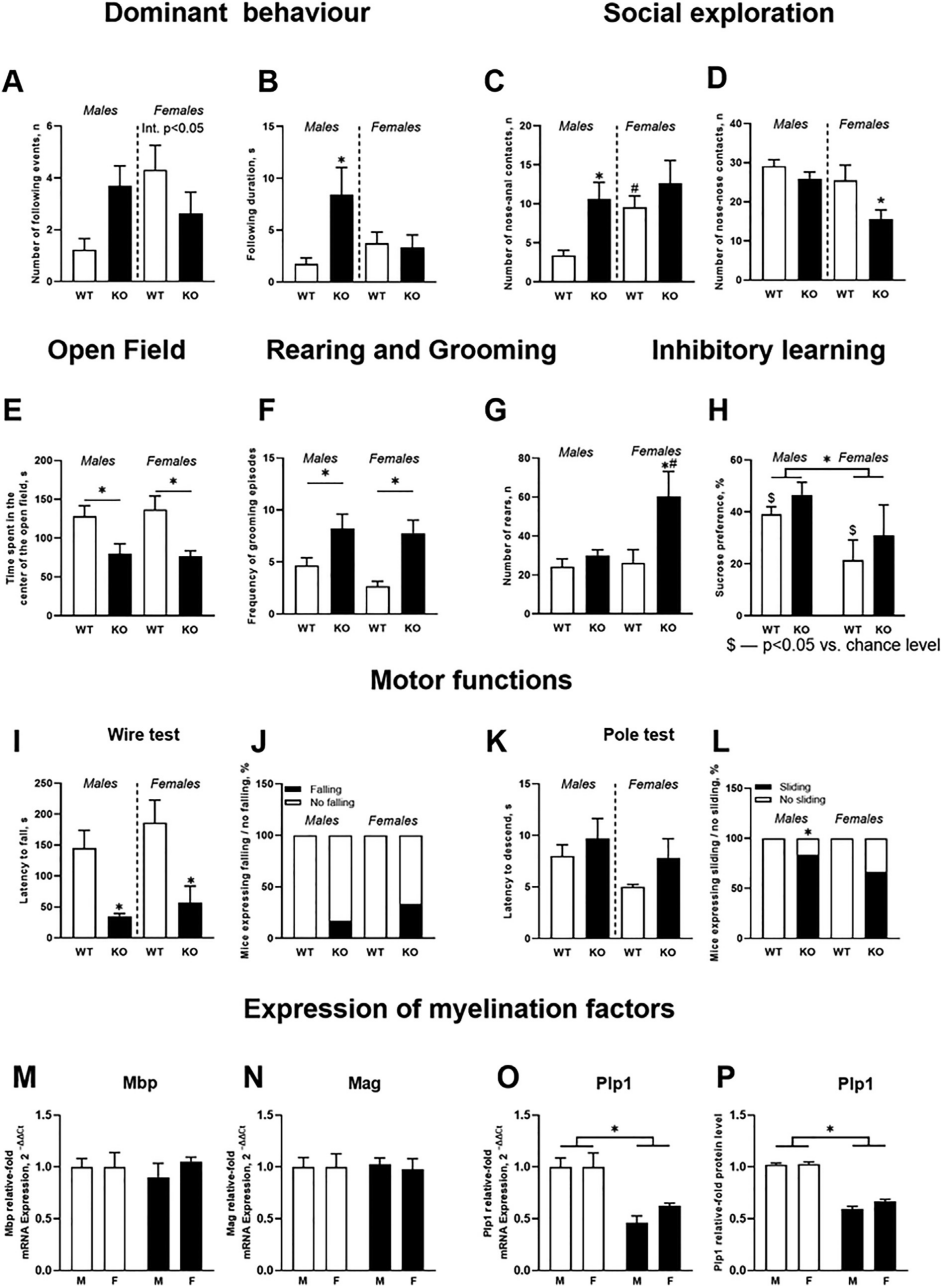
***St3gal5−/−* mice display behavioural and molecular abnormalities**. **(A**–**D)** Social behavior (n ​= ​14 in each group). **(E**–**G)** Anxiety-like and stereotypic behavior (n ​= ​10 per group for males, n ​= ​8 per group for females). **(H)** Associative inhibitory learning (n ​= ​6 in each group). **(I**–**L)** Motor functions (n ​= ​6 in each group). **(M**–**P)** Expression of mRNA of myelination transcripts and Plp1 protein (n ​= ​6 in each group). ∗p ​< ​0.05 vs. wild-type group, #p ​< ​0.05 vs. male group, $p ​< ​0.05 vs. 50%-chance level. ‘Genotype’ – significant effect of genotype. Two-way ANOVA and Tukey's test; one-sample *t*-test; Chi-square and Fisher's exact test; data present as Mean ​± ​SEM except **J** and **L** (*see the text*).

A two-way ANOVA revealed a significant sex×genotype interaction for the number of following events (p ​< ​0.05), but no group differences (p ​> ​0.05, Tukey's test; Fig. 1A). There was a significant sex×genotype interaction in the duration of following behavior (p ​< ​0.05), which was longer in male St3gal5−/− mice than in controls (p ​< ​0.05) and unaltered in females (p ​< ​0.05; Fig. 1B). There was a main effect of genotype on the latency to follow (p ​< ​0.05; Fig. 3A); mounting and attacking behaviors did not exhibit a sex×genotype interaction (Figs. S2B–G).

The number of nose-anal contacts exhibited a sex×genotype interaction (p ​< ​0.05, two-way ANOVA), this measure was higher in St3gal5−/−male mice than in wild type group (p ​< ​0.05, Tukey's test; Fig. 1C) that was not shown for females (p ​< ​0.05). Congruent changes were found in the duration of this behavior as suggested by a significant effect of genotype (Fig. S4A). There were no significant effects of sex, genotype, or their interaction on the latency to nose-anal contact (all p ​> ​0.05). Genotype significantly affected the duration of this behavior (p ​< ​0.05), which was increased in St3gal5−/− animals in comparison with controls irrespective of sex (both p ​< ​0.05, unpaired t-test, Fig. S4B). (All statistical values of behavioural analysis are presented in Table S2).

Only main effects of sex and genotype were significant for the number of nose-nose contacts (p ​< ​0.05, respectively, two-way ANOVA), where this measure was lower in mutants than in controls (p ​< ​0.05) and lower in females (p ​< ​0.05, Fig. 1D). Changes in the latency to nose-nose contacts, as well as other measures of social interaction were unchanged, except for the main effect of genotype on the duration of nose-nose contacts (p ​< ​0.05; Figs. 4C and D).

Two-way ANOVA showed significant effect of genotype, but not sex×genotype interaction for the time mice spent in the center of the open field (p ​< ​0.05 and p ​> ​0.05, respectively). This behavior was shorter in St3gal5−/− groups than in controls (p ​< ​0.05; Fig. 1E). The frequency of the grooming episodes in the open field center was significantly affected by genotype where it was increased in the mutants (p ​< ​0.05; Fig. 1F), but here there was no sex×genotype interaction (p ​< ​0.05 and p ​> ​0.05, respectively). While there was no sex×genotype interaction in the number of rears (p ​> ​0.05), there was a significant main effect of genotype and sex on this measure (both p ​< ​0.05), which was higher in female mutants than in controls and male mutants (both p ​< ​0.05; Fig. 1G).

During recall session of the conditioned taste aversion test, all PBS-treated groups and LiCl-treated mutants exhibited no significant difference in sucrose preference from the 50%-chance level (all p ​> ​0.05, one-sample t-test; Fig. 1H), while the LiCl-treated wild-type groups exhibited a decrease (p ​< ​0.05 for both males and females).

### 3.2 Motor deficits and lowered brain expression of myelin marker Plp1 in St3gal5−/− mice

Genotype significantly affected the latency to fall in the Wire test (p ​< ​0.05), which was shorter in St3gal5−/− mice than in controls (p ​< ​0.05; Fig. 1I). No group differences in the incidence of falling were observed (both p ​> ​0.05, Fisher's test; Fig. 1J). In the Pole test, we found increased incidence of sliding in male mutants (p ​< ​0.05) and no changes in the latency to descend (p ​> ​0.05; Fig. 1K,L). (Statistical values for the behavioural analysis and gene expression data are presented in Tables S2–S3).

Two-way ANOVA revealed a significant genotype effect on the concentration of Plp1 mRNA (p ​< ​0.05), but for the other myelin transcripts (all p ​> ​0.05; Fig. 1M,N, Fig. 5). In St3gal5−/− mice, the level of Plp1 mRNA and relative fold protein expression of Plp1 were lower than in controls (p ​< ​0.05; Fig. 1O,P); the latter measure was significantly affected by genotype only (p ​< ​0.05).

### 3.3 Altered behavioural and molecular response of St3gal5−/− mice to LPS

**Fig. 2:**
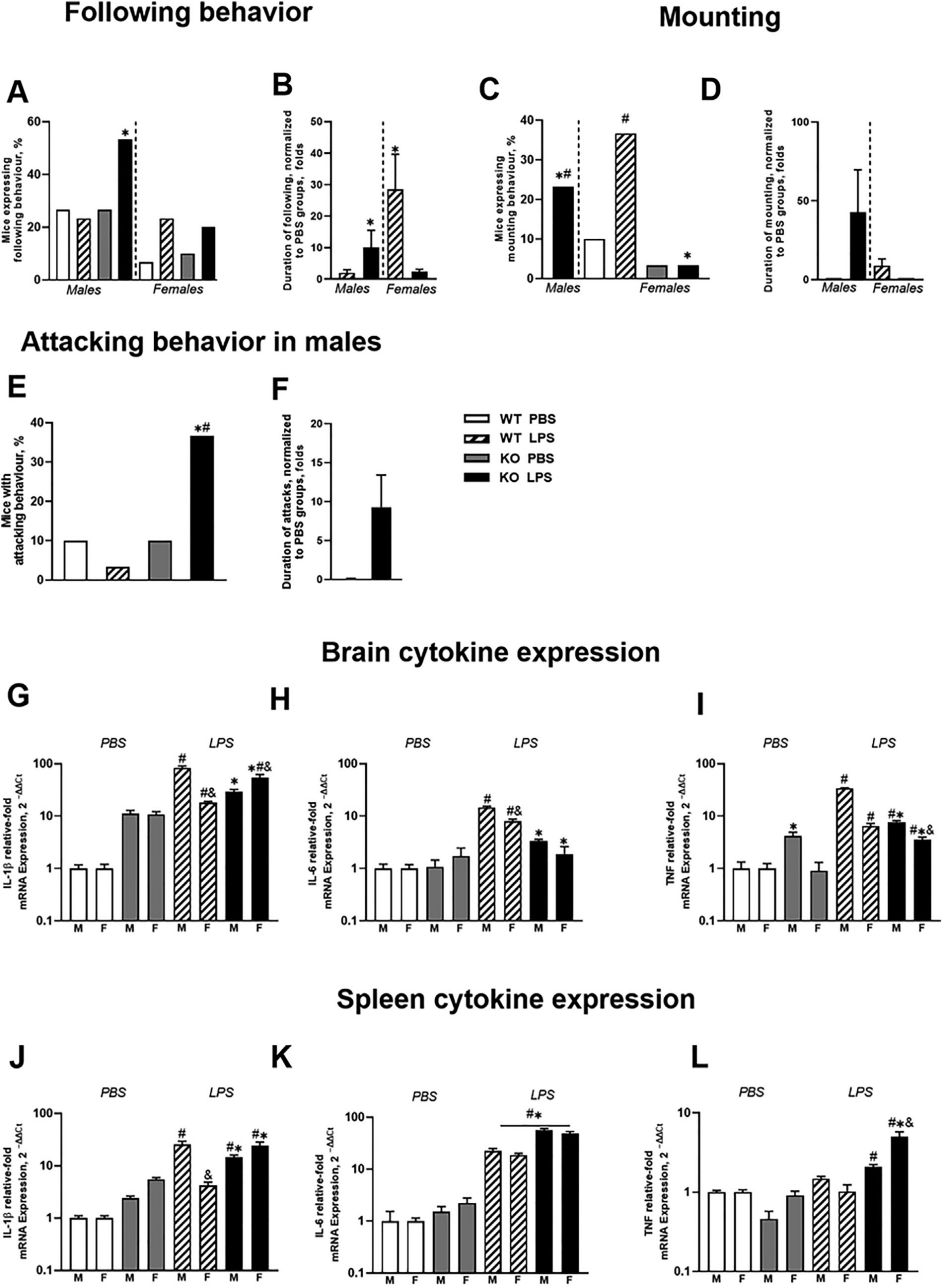
**Altered behavioural and molecular response of *St3gal5−/−* mice to LPS challenge. (A**–**F)** Dominant and aggressive behavior (n ​= ​6 in each group). **(G**–**I)** Expression of pro-inflammatory cytokines in the brain and **(J**–**L)** in the spleen, (n ​= ​4–6 in each group). ∗p ​< ​0.05 vs. wild-type group, #p ​< ​0.05 vs. PBS group. &p ​< ​0.05 vs male group. Int. — significant interaction. M - males, F - females. Two-way and three-way ANOVA, Tukey's and Sidak's post -hoc tests; Chi-square and Fisher's exact test; data present as Mean ​± ​SEM except **A** and **C** (*see the text*).

A Chi-square test revealed a significantly higher incidence of following in males St3gal5−/−-LPS-treated animals compared to the St3gal5−/−-PBS-treated and control-LPS-treated groups (both p ​= ​0.01, Fisher's test; Fig. 2A); no such differences were found in females (p ​> ​0.05). The duration of following was significantly affected by a sex ​× ​genotype interaction (p ​< ​0.05, two-way ANOVA). In comparison with the LPS-treated wild type groups, normalized to PBS-groups, the duration of following was unchanged in male LPS-treated mutants and decreased in the LPS-treated female mutants (p ​< ​0.05, Tukey's test; Fig.2B, Fig. S6A,B). However, no difference in the number of following episodes or the latency to follow was observed (Fig. S6A,B). (For statistical values of behavioural analysis, see Table S4).

There was a significant difference in the incidence of mounting in males and females (both p ​< ​0.01, Chi-square test). This behavior was not displayed by the PBS-treated male mutants. Among LPS-treated St3gal5−/− groups, the incidence was higher in males and lower in females compared to controls (both p ​< ​0.05; Fig. 2C). Normalized to the PBS-groups, the duration of mounting was unaltered in the LPS-treated mice (all p ​> ​0.05, two-way ANOVA; Fig. 2D). There was significant interaction, but no significant group differences in the number of mounting episodes or latency to mounting (Fig. S6C,D).

There was a significant difference in the incidence of attacks in the males (p ​< ​0.01, Chi-square test), which was s higher in the St3gal5−/−-LPS-treated group than in the St3gal5−/−-PBS treated group or the wild type LPS-treated mice (both p ​< ​0.05, Fisher's test; Fig. 2E). LPS-treated mutants exhibited an increased duration, normalized to PBS-groups, of attacks in comparison to St3gal5−/−-PBS group (p ​< ​0.05, *t*-test; Fig. 2F). No differences in the number of attacks or latency to attack were observed (Fig. S6E*,F*).

For brain expression of mRNA for cytokines IL-1β, IL-6 and TNF, significant sex ​× ​genotype ​× ​treatment interactions were observed following LPS administration (all p ​< ​0.01, three-way ANOVA). IL-1β mRNA and TNF mRNA concentrations were higher in LPS-treated animals than in PBS-treated groups (all p ​< ​0.05, Sidak's test; Fig. 2G). In the LPS-treated groups, IL-1β mRNA and TNF mRNA was lower in the *St3gal5−/−* males than in wild-type mice and higher in *St3gal5−/−* females than in controls and male mutants (all p ​< ​0.01). Wild-type mice, but not *St3gal5−/−* groups showed significant increases in IL-6 mRNA after LPS administration in comparison with PBS-treated groups (p<0.01 and p>0.05, for both males and females, respectively). LPS-treated mutants of both sexes had lower concentrations of IL-6 mRNA and TNF mRNA than LPS-treated wild-type mice (both p ​< ​0.01; Fig. 2H and I). In males, TNF mRNA levels were higher than in females among PBS-treated mutants and LPS-treated wild type and mutant mice (all p ​< ​0.01). (Statistical values for the gene expression data are presented in Supplementary Tables S5–7).

Spleen levels of IL-1β mRNA and TNF mRNA, but not of IL-6 mRNA were significantly affected by sex ​× ​genotype ​× ​treatment interaction (p ​< ​0.01, three-way ANOVA). LPS-treated groups, except in wild-type females, showed higher IL-1β mRNA concentrations (all p ​< ​0.01, Sidak's test; Fig. 2J). In the LPS-treated groups of mutants, IL-1β mRNA concentrations were lower in males and higher in females than in the wild type groups (both p ​< ​0.01). There was a significant interaction between treatment and genotype for spleen IL-6 mRNA concentrations (p ​< ​0.01). This measure was increased in all LPS-treated groups in comparison to the PBS-treated animals and was higher in *St3gal5−/−*groups than in the wild-type mice (all p ​< ​0.01, Tukey's test; Fig. 2K). Only mutants, both males and females, displayed a significant LPS-induced increase of TNF mRNA concentrations in comparison with PBS-treated groups; this increase was significantly higher in females (all p ​< ​0.01, Fig. 2L). (Statistical values for the gene expression data are presented in Supplementary Tables S5–7).

## 4 Discussion

We observed substantial abnormalities in dominant behavior and neutral sociability in the *St3gal5−/−* mice. The *St3gal5−/−* mutants also displayed impaired inhibitory learning, stereotypic features and moderate motor deficits, systemic inflammation and signs of hypomyelination. In addition, *St3gal5−/−* mutants exhibited aberrant behavioural and molecular responses to LPS. Naïve mutants showed decreased gene and protein expression of the major myelin component Plp1, and over-expression of pro-inflammatory cytokines in the brain and spleen. LPS administration to *St3gal5−/−*mice elicited increased dominant/attacking behaviours in males and decreased dominancy in females. Relative to the changes in wild-type mice, the expression of cytokines in the LPS-challenged *St3gal5−/−*mice was reduced in the brain and exacerbated in the spleen.

These abnormalities are reminiscent of the established phenotypes of ASD in clinic (Thapar et al., 2017) and in animal models (Haratizadeh et al., 2021). The Diagnostic and Treatment Manual for Mental Disorders, Fifth Edition (DSM-5) defines ASD by the expression of deficient social communication and interaction, restricted-repetitive behaviours and developmental impairments (DSM-5R, 2013). The core ASD-like characteristics in rodents can be recapitulated by the expression of aberrant reciprocal social behavior, stereotypy and cognitive deficits (Haratizadeh et al., 2021, Veniaminova et al., 2017, Veniaminova et al., 2020).

For example, in mice, abnormal interactions with counter partners, excessive following, dominancy, increased grooming and rearing, impaired inhibitory learning, are all signs of ASD-like behavior (Das et al., 2019; Lewis et al., 2007; Onore et al., 2013; Tatsukawa et al., 2019). We observed increased following behavior in male mutants, decreased social exploration in female mice, and aberrant nose-anal interaction in both sexes which are suggestive of social dysfunction. Social behaviours were also abnormally altered by the administration of LPS which is in keeping with findings that LPS exacerbates ASD-like behaviours in other mouse models including BTBR mice, a genetic model of ASD (Das et al., 2019; McFarlane et al., 2008; Onore et al., 2013). The impaired conditioned taste conditioning suggests cognitive rigidity in *St3gal5−/−* animals, an important element of ASD-like phenotype (Haratizadeh et al., 2021). The presence of motor deficits in the *St3gal5−/−* mice also agrees with other studies in which an association between ASD syndrome and motor dyscoordination has been described (Das et al., 2019; Lewis et al., 2007; Thapar et al., 2017).

Clinical studies suggest a pivotal role of altered immunity and response to systemic inflammation in the behavioural/cognitive signs of ASD (Ashwood et al., 2011; Jyonouchi et al., 2012; Napolioni et al., 2013). In comparison to controls displaying developmental disabilities other than autism, plasma levels of cytokines IL-1β, IL-6, IL-8 and IL-12p40 were increased in children with ASD, which correlated with the severity of the disease (Ashwood et al., 2011). The over-production of pro-inflammatory cytokines was also demonstrated by utilizing cultured and stimulated peripheral blood monocytes from children with ASD (Enstrom et al., 2010; Jyonouchi et al., 2012). Over-expression of IL-1β, IL-6, IL-17 and TNF has also been reported in the adult autistic brain (Napolioni et al., 2013; Theoharides et al., 2016; Wei et al., 2013).

Our data showing central and peripheral over-production of IL-1β, IL-6, and TNF are in keeping with these findings, as well as with previous demonstrations of systemic inflammation and up-regulated brain expression of IL-1β, IL-6, IL-17, IL-18, IL-33, TNF and astrocyte/microglia activation in animal models of ASD (Das et al., 2019; Prata et al., 2017; Wei et al, 2012, 2016). We also found the upregulation of IL-1β in the double transgenic *St3gal5-*deficient 5xFAD female mice (Dukhinova et al., 2019). The synergistic interaction of abnormal baseline production of IL-1β, IL-6 and TNF in mutants and LPS-induced systemic inflammation is reminiscent of a ‘double hit’ phenomenon (Carlezon et al., 2019; Couch et al, 2013, 2016; Onore et al., 2013; Yasumatsu et al., 2020) and could explain the exacerbation of the changes in social behavior observed in the *St3gal5−/−* mice. At the same time, no differences were fund in counts for mononuclear and polymorphonuclear white blood cells between controls and mutants (Ponomarev, *unpublished results*). The current study also revealed the presence of muted responses in cytokine expression following LPS in the mutants. This is in keeping with observations that cytokine production is reduced in the monocytes of children with ASD after TLR-targeted stimulation (Jyonouchi et al., 2012) and with decreased brain expression of IL-6 and TNF in *St3gal5−/−* mice after brain injury (Dukhinova et al., 2018).

Sex-specific expression profiles of TNF, TGF-β, IFN-γ, IL-17 and IL-6 were recently reported in ASD patients (Eftekharian et al., 2018). *St3gal5−/−*mice display a sex bias in cytokine expression and this bias may underlie the sex-dependent behavioural changes reported here and elsewhere (Niimi et al., 2011). Similarly, LPS-induced social and repetitive behaviours in C57BL6 mice were sex-dependent (Carlezon et al., 2019).

Pro-inflammatory changes in mutants are likely to be owing to the lack of gangliosides of the a-, b- and c-series, which exert anti-inflammatory functions (Schnaar, 2016). This imbalance is, seemingly, not compensated by expression in the *St3gal5−/−* mice of GD1α and GM1b, whose role in inflammation has been not shown (Trinchera et al., 2018). Congruently, signs of deficit in myelination, which is regulated by a- and b-series gangliosides, is unlikely to be compensated by the 0-gangliosides in the *St3gal5−/−* mutants. The decreased Plp1 expression in the *St3gal5−/−* mice is in keeping with the myelin changes observed in carriers of *St3gal5* gene variants that are associated with ganglioside deficiency (Bowser et al., 2019). Decreased Plp1 expression may also result from a bi-directional relationship with neuroinflammatory processes (Groh et al., 2018), that can compromise synaptic plasticity, brain connectivity, and motor function (Thapar et al., 2017), as well as give rise to aberrant social behavior. For example, studies have linked hypomyelination in the medial prefrontal cortex with behavioural abnormalities in a mouse ASD model (Makinodan et al., 2017).

Thus, genetic deficiency in a-, b- and c-series of ganglioside in the brain, due to the genetic lack of ST3GAL5 enzyme, leads to the abnormalities that are reminiscent to the ASD-like syndrome. We would not argue that gangliosides deficiency is likely to be a causal element of ASD pathology, but it could be a contributing factor in some cases. Our work warrants future investigation into the potential link between ASD and deficits in brain glycoprotein sialylation and a- and b-series ganglioside synthesis. In particular, further studies are needed to examine the correlation between the molecular and behavioural changes, whether the ASD-like phenotype can be recovered with a therapeutic intervention, and to better understand the impact of the myelin associated changes. Yet, given ASD is a disorder of highly variable genetics with as-yet-unknown pathophysiology, the *St3gal5−/−* mice provide a useful animal model to explore ASD-like features associated with ganglioside deficiency.

## Declaration of competing interest

On behalf of all authors, I would like to state that none of the authors involved in the work have any competing interest.
