# Supplementary material for: ASD-like behaviors, a dysregulated inflammatory response and decreased expression of PLP1 characterize mice deficient for sialyltransferase ST3GAL5
Source: Brain Behav Immun Health. 2021 Jul 27;16:100306. doi: 10.1016/j.bbih.2021.100306 (PMC8474501; doi:10.1016/j.bbih.2021.100306)
Supplement: Multimedia component 1 [file mmc1.docx]

Supplementary File

**Methods**

**Animals**

Eight to twelve-week old male and female *St3gal5−/−* mice or C57BL/6 mice were used. Five-week-old counter partners in the social interaction test were also employed (Dukhinova et al., 2019, 2018; Sotnikov et al., 2013). Male mice were housed individually in standard plastic cages (27x22x15 cm), and female mice were housed in groups of three-five per cage. Mice were maintained on reversed 12-h light/dark cycle, under controlled laboratory conditions (22±1°C, 55% humidity, room temperature 22ºC, lights were on at 19:00), and food and water were available ad libitum. All efforts were undertaken to minimize discomfort and experimental protocols conformed to 2010/63/EU and were compliant with the ARRIVE guidelines (“ARRIVE guidelines,” n.d.). The experiments were approved by local veterinarian committee of School of Biomedical Sciences, Faculty of Medicine, at the Chinese University of Hong Kong, Shatin, Hong Kong.

**Study design**

The social behaviour of the *St3gal5−/−* mice was studied in two sets of experiments (Fig. S1A&B). In cohort A, mice were first employed to examine social interaction and rearing behaviours (n=14 in each group) and then they were used to detect the ability to induce conditioned taste aversion (n=6 in each group; Fig.S1A). In cohort B, the mice were used to investigate social behaviour or cytokine expression in following the i.p. administration of LPS (0.1mg/kg) or PBS (n=6 per group). Additional groups of mice were used to study behaviour in the open field and grooming (n=10 per group for males, n=8 per group for females). The motor function of *St3gal5−/−* mice and wild-type animals was evaluated in the Pole test and Wire test (n=6 in each group). The mRNA levels for myelin proteins and protein concentrations of Plp1 in the brain cortex were studied in separate cohorts (n=3-6). For all protocols used, *see below.*

| 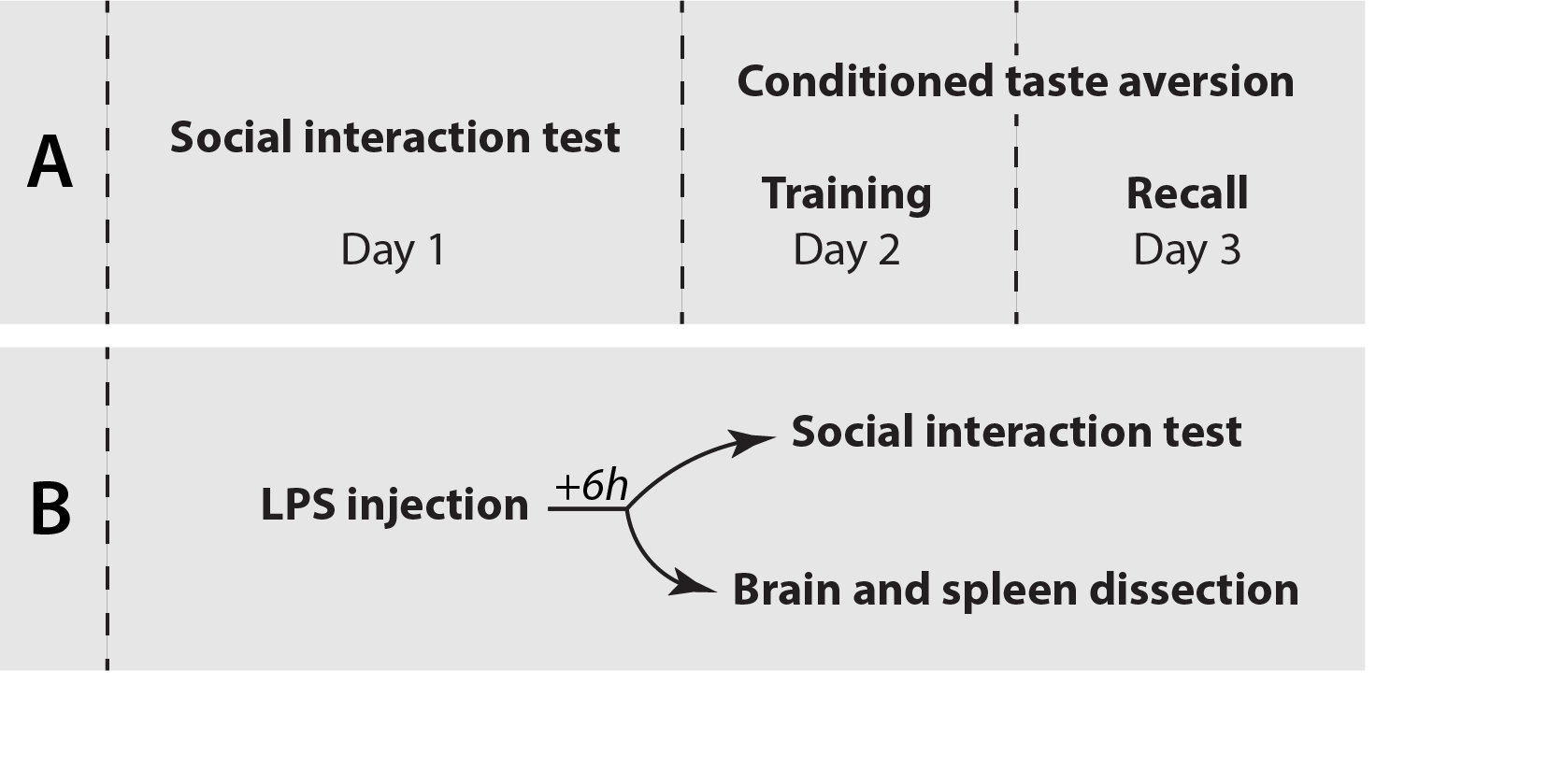 |
| --- |
| **Figure S1. Experimental design of the studies containing a social interaction test. (A)** St3gal5−/− and wild-type mice of both sexes were tested in the social test on day 1, and in the conditioned taste aversion on days 2-3. **(B)** St3gal5−/− and wild-type mice of both sexes with LPS or PBS. After 6 hours post administration, half of the animals were studied in the social interaction test, and other half were killed, the brain and spleen were collected for subsequent RT PCR assay. |

**Behavioral tests**

Behavioral tests were carried out during an active period of the animals' light cycle (09:00–21:00) and analyzed offline by the experimenter who was unaware of the genotype of each animal studied. One test was run per day. Mice of both genotypes were tested simultaneously. Behavioural equipment was thoroughly cleaned with water between each test.

*Social interaction test*

The social interaction test was adapted from a previously described method (Couch et al., 2016; Gorlova et al., 2020, 2019; Veniaminova et al., 2017). Each mouse was habituated to a new plastic cage (43 x 27 x 19 cm) in which an unfamiliar group-housed naïve juvenile mouse of the same sex, at five-weeks old, was placed for 10 min. The latency, total duration, and number of events of aggressive (attacking) behaviour and dominant-like behaviours: following and mounting, and two forms of neutral social exploration: nose-nose and nose-anal contact were scored. In the study with LPS, the number of mice exhibiting dominant-like behaviour and neutral social exploration during two-minute intervals was recorded.

*Conditioned taste aversion*

During the training session, mice were deprived from water between 17.00 and 14.00 (for 21 h) and were then allowed to drink a 2.5% sucrose solution for 30 min in one-bottle paradigm (Strekalova, 2008; Strekalova and Steinbusch, 2009). Thereafter, the mice received an intraperitoneal injection of solution of lithium chloride (LiCl, 0.24 M) at the dose 2% of body weight or PBS. After the injection, the animals were allowed to have access to a sucrose solution for 1.5 h and thereafter they were subjected to water deprivation for 12 h. On the next day, a test for recall was performed. Mice were given a choice between tap water or 1% a sucrose solution in a two-bottle paradigm for 8h. The amount of liquid consumed was determined by weighing the bottles before and after a drinking session, and the preference to sucrose solution was calculated according to the following formula:

Sucrose preference=100%×(Amount of sucrose solution consumed, g)/(Total amount of liquid consumed, g)

A decrease of sucrose preference during the recall session in comparison with a chance level is considered as a sign of taste aversion, i.e., inhibitory associative learning.

*Wire test*

The Wire test was used to assess motor function in mice as previously described (de Munter et al., 2020; Veniaminova et al., 2020). Mice were allowed to grip onto a horizontal hanging wire (0.3 cm in diameter at 60 cm above the surface) using their four limbs, and the latency to fall was scored in two consecutive sessions and the mean was calculated. The incidence of falling events was also recorded.

*Pole test*

Mice were placed on top of a vertically standing bar (diameter 1.1 cm, height 60 cm) and allowed to climb down to a horizontal surface as described elsewhere (de Munter et al., 2020; Veniaminova et al., 2020). The latency to descend, measured as time spent by the animal to reach the ground with all four paws. Sliding events were also recorded as a measure of motor function in the two trials.

*Open field test*

Mice were placed in the central arena of a customized square open field (40 x 40 cm), which was illuminated with a red light. Their behaviour was video recorded for 10 min as described elsewhere (Lim et al., 2016). The time spent in the central zone (30 x 30 cm), and duration of grooming were analysed offline using Any-maze software (Anymaze, Dublin, Ireland).

**Brain dissection**

Brain cortex was harvested as described elsewhere (Dukhinova et al., 2018; Kopeikina et al., 2020). For brain cortex dissection, the brains were isolated on ice, olfactory bulbs and cerebellums were removed (see Fig. S2) and the overlying cortex was removed from the subcortical structures.

|   **Figure S2. A scheme of cortex dissection.** |
| --- |

**Real-time polymerase chain reaction (RT-PCR)**

qRT-PCR was performed using the SYBR Green master mix (Bio-Rad Laboratories, Philadelphia, PA, USA). qRT-PCR was performed in a 10μl reaction volume containing a SYBR Green master mix (5 ul), RNase-free water (3 μl), specific forward and reverse primers used at the concentration 20 pmol/ul (1 μl) and cDNA (1 μl). The initial denaturation step for qRT-PCR was performed at 95°C for 5 min followed by 40 cycles of denaturation at 95°C for 30 seconds and annealing at 60°C for 30 seconds. The sequences of primers used are listed in Table S1 (see below); all primers were purchased from Life Technologies (Carlsbad, CA, USA). All samples were run in triplicate.

**Table S1. Primer sequence for mRNA expression analysis**

| **Gene** | **Primer** | **Sequence** |
| --- | --- | --- |
| **Plp1** | Forward | 5’-CCAGAATGTATGGTGTTCTCCC-3’ |
|  | Reverse | 5’-GGCCCATGAGTTTAAGGACG-3’ |
| **Mbp** | Forward | 5’- TCACAGCGATCCAAGTACCTG-3’ |
|  | Reverse | 5’-CCCCTGTCACCGCTAAAGAA-3’ |
| **Mag** | Forward | 5’-GGTACATGGCGTCTGGTATTTC-3’ |
|  | Reverse | 5’-ACTTGTGTGCGGGACTTGAAG-3’ |
| **Mog** | Forward | 5’-TCATGCAGCTATGCAGGACAA-3’ |
|  | Reverse | 5’-TTTCGGTAGAGGTGAACCACT-3’ |
| **IL-1β** | Forward | 5’- CTTCCAGGATGAGGACATGAGCAC -3’ |
|  | Reverse | 5’-TCATCATCCCATGAGTCACAGAGG -3’ |
| **IL-6** | Forward | 5’-CCTTCTTGGGACTGATGCTGGTG-3’ |
|  | Reverse | 5’- AGGTCTGTTGGGAGTGGTATCCTC-3’ |
| **TNF** | Forward | 5’-AGCCGATGGGTTGTACCTTG- 3’ |
|  | Reverse | 5’- GTGGGTGAGGAGCACGTAGTC -3’ |
| **GAPDH** | Forward | 5’-ATGACCACAGTCCATGCCATC -3’ |
|  | Reverse | 5’-GAGCTTCCCGTTCAGCTCTG -3’ |

**Western blotting**

Samples of brain cortex (0.1 g of wet tissue) were lysed in 1 ml of lysis buffer (50 mM of Tris-HCl (pH=7.4), 150 mM of NaCl, 2%SDS, 1% Sodium Deoxycholate, 5 mM EDTA, 1% NP40 (Tergitol Solution, Sigma, St. Louis, MO, USA), and 1:100 protease inhibitor cocktail (cat#1860932, Thermo Fisher Scientific Hong Kong, Hong Kong), and lysates were passed through 18G needle 10 times, vortexed for 5 min and centrifuged at 10,000 rpm for 10 min at 4C. The supernatant was collected and 5 µl of supernatant and was mixed with 195 µl of Laemmli buffer. Then 10 µl of samples were loaded onto SDS electrophoresis gel (BoltTM 8% Bis-Tris Plus Mini Gels, Ref. NW00082BOX, Invitrogen, Thermo Fisher Scientific Hong Kong, Hong Kong) according to manufacturer’s recommendations using BoltTM MES SDS Running buffer, at a voltage of 200V for 25 minutes. Immunoblotting was performed using Imobilon-PSQ polyvinylidene difluoride (PVDF) membranes (cat# ISEQ00010, Millipore, Bedford, MA, USA). PVDF membranes were incubated in a 99%-methanol solution for 1 min (Sigma, St. Louis, MO, USA), then washed with mQ H2O for 5 min, and then incubated in Tris/Glycine transfer buffer (cat#161-0734, Bio-Rad Pacific Limited, Hong Kong) for 15 min. Transfer was performed on ice using ice-cold transfer buffer with constant voltage 80V for 2h. After transfer, the membrane was treated with a 5% BSA the TBST, containing 50 mM Tris-HCl (pH=8.2), 150 mM NaCl, 0.05% Tween-20 (Sigma, St. Louis, MO, USA) for 1 h at the room temperature and subsequently incubated with antibodies to Plp1 (1:1000, cat#ab28486, Abcam, Cambridge, U.K.) and β-Actin (1: 1000, cat#4967; Cell Signalling, Beverly, MA, USA) at 4°C overnight that was followed by the incubation with secondary horseradish peroxidase-conjugated secondary (HRP) antibodies (1:1000, cat#7074S, Cell Signaling, Beverly, MA, USA), for 1h at room temperature on a rotary shaker. Bands were visualized using Peroxide Detection Reagent from ECL Substrate kit (cat#ab133406, Abcam, Cambridge, U.K.) according to manufacturer’s recommendations. β-Actin was used as a loading control. Quantitative analysis of relative expression levels of PLP1 was normalized to β-Actin as we reported earlier (Veremeyko et al., 2018).

**Results**

**Dominant and aggressive behavior**

No significant interaction between sex and genotype was found for the latency to follow (p=0.34, two-way ANOVA). A significant main effect of genotype was observed (p<0.05, two-way ANOVA). Latency to follow in the wild-type mice was significantly higher compared to *St3gal5−/−* groups (p=0.02, unpaired t-test). No significant differences in the latency to mount, the number of mounting events, and mounting duration were observed (all p>0.05, two-way ANOVA). In males, no significant differences were found in the latency to attack, the number, or duration of attacks.

(Statistical values of behavioral analysis are also presented in *Table S2*).

|  |
| --- |
| **Figure S3. Dominant and aggressive behaviours. (A)** Latency to following was significantly lower in *St3gal5−/−* animals irrespective of sex. **(B-D)** Parameters of mounting and **(E-F)** parameters of attacks in males were not changed significantly. WT — wild type. KO — *St3gal5−/−.*  Data are Mean ± SEM. |

**Neutral social exploration**

The duration of both nose-anal and nose-nose contacts was affected by the genotype alone (both p<0.05, two-way ANOVA, Fig.S4). No significant changes in the latencies to nose-anal and nose-nose contacts were revealed by two-way ANOVA.

|  |
| --- |
| **Figure S4. Neutral social exploration in *St3gal5−/−* mice. (A)** Duration of nose-anal contacts. **(B)** Latency to nose-anal contact. **(C)** Duration of nose-nose contacts. **(D)** Latency to nose-nose contact. WT — wild type. KO — *St3gal5−/−.* Data are Mean ± SEM.  **RT-PCR and Western blotting**  There were no significant differences in the expression of Mog (all p>0.05, two-way ANOVA, Fig.S5A). Plp1 relative-fold protein expression was significantly affected by genotype alone (p<0.01, two-way ANOVA, Fig. S5B). A significant decrease of Plp1 relative-fold protein expression was found in the *St3gal5−/−* mice in comparison to wild-type controls. (Statistical values of Western blot analysis are also presented in *Table S3).*  **A B** |


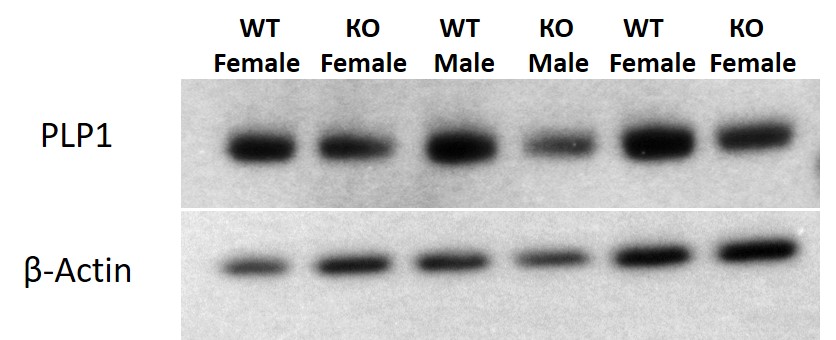


**Figure S5 (A) Expression of Mog in the brain cortex of *St3gal5−/−* mice.** No significant group differences in the levels of MogmRNA were found in wild-type and ***St3gal5−/−*** mice (p>0.05; two-way ANOVA). Data are Mean ± SEM. **(B)** Representative image of Western blot analysis of PLP1 expression in the brain of wild-type and ***St3gal5−/−*** mice. WT — wild type. KO — *St3gal5−/−.*

**Changes in social behaviors in the LPS-treated mice**

**Aggressive and dominant behavior**

No significant differences in the latency to a following events. Was noted (all p>0.05, two-way ANOVA, Fig. S6A). For the latency to a mounting event, three-way ANOVA revealed a significant sex × genotype interaction (p=0.01, Fig. S6B). *St3gal5−/−* female mice exhibited a significantly increased latency to mounting events compared to wild-type female group irrespective of treatment (LPS) (p=0.04, unpaired t-test). Significant main effects of treatment, genotype, and sex were found for the number of following events (all p<0.05, three-way ANOVA. Fig.S6C). A significant sex × genotype × treatment interaction was also found for the number of mounting events (p<0.05, three-way ANOVA. Fig. S6D). Female LPS-treated wild-type group exhibited a significantly higher number of mounting events than the LPS-treated wild-type male group and the *St3gal5−/−* LPS-treated female group (both p<0.05, Sidak’s test). A interaction between treatment and sex significantly affected the duration of following (p<0.05, three-way ANOVA. Fig.S6E). Irrespectively of genotype, LPS-treated female groups exhibited an increased duration of following compared to LPS-treated males, and same effect was observed in PBS-treated groups (both p<0.05, Tukey’s test). The duration of mounting was significantly affected by an interaction between sex, genotype, and treatment (p<0.05, three-way ANOVA. Fig.S6F). Compared to the control-LPS-treated male group and the *St3gal5−/−-*LPS-treated female group, female LPS-treated wild-type group exhibited a significantly increased duration of mounting (both p<0.05, Sidak’s test) In male mice, no significant differences were found for the number of attacks or in the latency to attack in the social interaction test (all p>0.05, three-way ANOVA, Fig.S6G,H). Compared to the control-LPS-treated male group and the *St3gal5−/−* -LPS-treated female group, female LPS-treated wild-type group exhibited a significantly increased duration of mounting (both p<0.05, Sidak’s test) In male mice, no significant differences were found for the number of attacks or in the latency to attack in the social interaction test (all p>0.05, three-way ANOVA, Fig.S6G,H). (Statistical values of behavioral analysis are also presented in *Tables S5A,B*.)

**Figure S6. Aggressive and dominant behavior in LPS-treated groups, normalized to the wild-type PBS groups.** (A) Latency to follow was not affected significantly by any of the factors. **(B)** A significant sex × genotype interaction was found for the latency to mount**. (C)** The number of following events was significantly affected by the main effects of sex, genotype, and treatment, but not by their interactions. **(D)** The number of mounting events was higher in LPS-treated wild-type female mice, than in the LPS-treated mutant female mice, and the LPS-treated wild-type male mice. **(E)** The duration of following exhibited a treatment × sex interaction. **(F)** Duration of mounting events was elevated more in LPS-treated wild-type female mice than in the LPS-treated mutant female mice, and in the LPS-treated wild-type male mice. **(G)** The number of attacks and **(H)** the latency to attack in males were not affected by either genotype, sex, or treatment. WT — wild type. KO — st3gal5−/−. Int. — interaction. *p<0.05 vs. wild type group, &p<0.05 vs. group of males. Data are Mean ± SEM.

**Neutral social exploration**

No significant differences were found by three-way ANOVA in the latency to a nose-anal contact, or in the latency, duration, or number of nose-nose contacts (all p>0.05, three-way ANOVA. Fig.S7A,B,D,F). For the duration of the nose-anal contacts, three-way ANOVA revealed a significant effect of the genotype (p=0.04. Fig.S7C). Irrespective of the sex and LPS treatment, st3gal5−/− mice showed enhanced duration of the nose-anal contacts. The number of nose-anal contacts also was found to be significantly affected by the genotype alone (p<0.05, Fig.S7E). *St3gal5−/−* mice exhibited significantly increased number of the nose-anal contacts compared to wild-type mice irrespectively of sex and treatment.

(Statistical values of behavioral analysis are presented in *Tables* *S6A,B*).

**Expression of the cytokines in the brain and the spleen normalized to sex- and genotype-matched PBS groups**

Two-way ANOVA revealed significant sex × genotype interaction for the expression of IL-1β mRNA, IL-6 mRNA, and TNF mRNA in the brain of LPS-treated mice (all p<0.01, two-way ANOVA. Fig.S8A,B). In both wild-type male and female mice an increase in the concentration of the IL-1β mRNA in the brain after LPS administration was significantly higher than in the corresponding *St3gal5−/−-*LPS groups (both p<0.05, Tukey’s test). In male wild-type mice, the increase of this measure was significantly higher than in wild-type group of females (p<0.01, Tukey’s test). In *St3gal5−/−* -LPS-treated mice, an increase in the IL-1β mRNA concentration upon LPS administration was more pronounced in female mice, compared to *St3gal5−/−-*LPS-treated males (p<0.01, Tukey’s test). LPS-induced augmentation of brain concentration of IL-6 mRNA was greater in the wild-type LPS-treated groups than in *St3gal5−/−* animals (both p<0.01, Tukey’s test). In both genotypes such increases were significantly higher in male animals than in female groups (both p<0.01). Similar increases were found for the brain concentration of TNF mRNA, whose increases were higher in the wild-type groups than in the mutants (both p<0.05, Tukey’s test). In male wild-type-LPS-treated animals, the elevation in concentration of TNF mRNA in the brain was higher than in wild-type-LPS-treated female mice (p<0.01, Tukey’s test), and opposite changes were found in the *St3gal5−/−* groups (p<0.01, Tukey’s test). (Statistical values are presented in *Tables S5-7*).

|  |
| --- |
| **Figure S7. Social interaction in LPS-treated groups. (A, B)** No significant differences were found in the latencies to nose-anal or nose-nose contacts, as well as in the **(D)** duration and **(F)** number of nose-nose contacts. **(C, E)** In the duration and number of nose-anal contacts, only genotype effect was significant. per group. WT — wild type. KO — *St3gal5−/−*. **M** — male mice, **F** — female mice. Int. — interaction. *p<0.05. Data are Mean ± SEM. |

In the spleen, IL-1β mRNA and IL-6 mRNA were significantly affected by the interaction of sex and genotype (both p<0.05, two-way ANOVA. Fig.S8C,D). Elevation of IL-β mRNA concentration following LPS administration was significantly higher in wild-type males compared to *St3gal5−/−* males and wild-type females (both p<0.01, Tukey’s-test). In the wild-type-LPS-treated female group, increase in the IL-6 mRNA concentration was significantly higher in comparison to both male wild-type-LPS group and female *St3gal5−/−* -LPS-treated animals (both p<0.01, Tukey’s-test). For the TNF mRNA concentration in the spleen, only genotype had a significant main effect (p<0.01, two-way ANOVA). Elevation in the concentration of the TNF mRNA in the spleen in the *St3gal5−/−* -LPS-treated groups was significantly higher than in the wild-type-LPS-treated groups irrespectively of the sex. (Statistical values are presented in *Tables S5-7*).

| **Figure S8. Expression of pro-inflammatory cytokines normalized to sex-and genotype-matched groups. (A, B)** All the LPS-treated groups of both sexes showed a significant elevation of the mRNA concentrations of the cytokines in the brain. In mutant males, elevation in the concentrations of IL-1β mRNA and TNF mRNA were lower compared to wild-type males. The same was found for the IL-6 mRNA and TNF mRNA in the female groups. IL-1β mRNA concentration in females exhibited changes that were opposite to those observed in the males. **(C,D)** In the spleen of LPS-treated mice, mRNA concentrations of all the cytokines were elevated significantly in comparison to PBS-treated groups, except TNF in wild-type females. In male mice, IL-1β and TNF mRNA were elevated significantly higher in wild-type-LPS mice in comparison to the *St3gal5−/−-*LPS animals. IL-6 in males and all three cytokines in females were significantly elevated in LPS-treated mutants vs. LPS-treated wild-type animals in the spleen. WT — wild type. KO — st3gal5−/−. *p<0.05 vs. PBS group, *p<0.05 vs. wild-type group. Data are Mean ± SEM. |
| --- |
| **Statistical analysis** |

***Table S2***. ***Summary of comparisons between wild type and St3gal5−/− male and female groups in the measures of behavior (two-way ANOVA and post-hoc Tukey’s test; Chi-square and Fisher’s exact test). Fig 1. A-L, Fig. S3-S4***

| **Two-way ANOVA** | | | | | | | | | | |
| --- | --- | --- | --- | --- | --- | --- | --- | --- | --- | --- |
| *Behavior* | *Parameter* | *Interaction, F* | *Interaction, p* | | *Genotype, F* | | *Genotype, p* | | *Sex, F* | *Sex, p* |
| ***Dominant and aggressive behavior*** | | | | | | | | | | |
| Following | Latency | 0.96 | 0.34 | | **4.35** | | **<0.05** | | 1.22 | 0.28 |
|  | Duration | **5.48** | **0.02** | | **4.18** | | **<0.05** | | 1.02 | 0.32 |
|  | Number | **5.47** | **0.02** | | 0.19 | | 0.66 | | 1.27 | 0.26 |
| Mounting | Latency | 1.49 | 0.23 | | 0.21 | | 0.65 | | 0.34 | 0.56 |
|  | Duration | 0.35 | 0.56 | | 0.43 | | 0.52 | | 1.78 | 0.19 |
|  | Number | 0.12 | 0.79 | | 0.06 | | 0.81 | | 3.81 | 0.06 |
| ***Neutral social exploration*** | | | | | | | | | | |
| N-A contact | Latency | 0.60 | 0.45 | | 0.04 | | 0.85 | | 3.24 | 0.09 |
|  | Duration | 0.47 | 0.50 | | **5.65** | | **0.02** | | 0.76 | 0.39 |
|  | Number | **22.63** | **<0.01** | | **11.65** | | **<0.01** | | **15.12** | **<0.01** |
| N-N contact | Latency | 0.61 | 0.44 | | 1.20 | | 0.28 | | 0.53 | 0.47 |
|  | Duration | 0.70 | 0.41 | | **12.49** | | **<0.01** | | 0.46 | 0.50 |
|  | Number | 1.32 | 0.26 | | **4.99** | | **0.03** | | **5.53** | **0.02** |
| ***Open field and repetitive behavior*** | | | | | | | | | | |
| Open field | Center time | 0.15 | 0.70 | | **13.2** | | **<0.01** | | 0.02 | 0.87 |
| Grooming | Frequency | 0.7 | 0.41 | | **21.82** | | **<0.01** | | 1.71 | 0.20 |
| Rears | Number | 3.70 | 0.06 | | **4.83** | | **0.03** | | **7.44** | **<0.01** |
| ***Aversive memory formation*** | | | | | | | | | | |
| Sucrose preference in the CTA | | 0.02 | 0.89 | | 1.23 | | 0.28 | | **4.85** | **0.04** |
| ***Motor functions*** | | | | | | | | | | |
| Wire test | Latency to fall | 0.14 | 0.71 | | **20.83** | | **<0.01** | | 1.41 | 0.24 |
| Pole test | Latency to descend | 0.16 | 0.70 | | 2.35 | | 0.14 | | 2.71 | 0.12 |
| **Tukey’s tests** | | | | | | | | | | |
| *Behavior* | *Parameter* | *WT ♂ vs ♀, p* | | *KO ♂ vs ♀, p* | | *♂ KO vs WT, p* | | *♀ KO vs WT, p* | | |
| ***Dominant and aggressive behavior*** | | | | | | | | | | |
| Following | Latency | >0.99 | | 0.46 | | 0.07 | | 0.90 | | |
|  | Duration | 0.79 | | 0.09 | | **0.03** | | >0.99 | | |
|  | Number | 0.09 | | 0.82 | | 0.31 | | 0.41 | | |
| Mounting | Latency | 0.97 | | 0.58 | | 0.92 | | 0.72 | | |
|  | Duration | 0.54 | | 0.95 | | 0.73 | | >0.99 | | |
|  | Number | 0.68 | | 0.37 | | >0.99 | | 0.98 | | |
| ***Neutral social exploration*** | | | | | | | | | | |
| N-A contact | Latency | 0.89 | | 0.29 | | 0.90 | | 0.98 | | |
|  | Duration | >0.99 | | 0.68 | | 0.07 | | 0.72 | | |
|  | Number | **<0.01** | | 0.93 | | **<0.01** | | 0.72 | | |
| N-N contact | Latency | >0.99 | | 0.71 | | 0.42 | | >0.99 | | |
|  | Duration | 0.60 | | >0.99 | | 0.25 | | **0.02** | | |
|  | Number | 0.84 | | 0.07 | | 0.90 | | **<0.05** | | |

***Table S2*** ***(continued)***

| ***Open field and repetitive behavior*** | | | | | | | |
| --- | --- | --- | --- | --- | --- | --- | --- |
| Open field | Center time | 0.97 | >0.99 | | | 0.11 | **0.04** |
| Grooming | Frequency | 0.35 | 0.99 | | | 0.06 | **<0.01** |
| Rears | Number | >0.99 | **0.03** | | | 0.94 | **0.01** |
| ***Inhibitory learning*** | | | | | | | |
| Sucrose preference in the CTA | | 0.37 | 0.48 | | | 0.90 | 0.81 |
| ***Motor functions*** | | | | | | | |
| Wire test | Latency to fall | 0.70 | 0.94 | | | **0.02** | **<0.01** |
| Pole test | Latency to descend | 0.49 | 0.81 | | | 0.85 | 0.53 |
| **Fisher’s exact test** | | | | | | | |
| *Parameter* | *Males, p* | | | *Females, p* | | | |
| Incidence of falling | >0.99 | | | 0.45 | | | |
| Incidence of sliding | **0.01** | | | 0.06 | | | |
| **Unpaired t-test (attacks, males)** | | | | | | | |
| *Parameter* | *t, df* | | | | p | | |
| Duration of attacks | t=0.9785, df=19 | | | | 0.34 | | |
| Number of attacks | t=0.6429, df=19 | | | | 0.53 | | |
| Latency to attack | t=0.4683, df=19 | | | | 0.64 | | |

**Table S3. Summary of comparisons in expression of myelination markers in the brain of wild type and St3gal5−/− male and female mice (two-way ANOVA and post-hoc Tukey’s test). Fig. 1 M-P, Fig. S5.**

| ***Expression of myelination proteins*** | | | | | | | | | | | |
| --- | --- | --- | --- | --- | --- | --- | --- | --- | --- | --- | --- |
| **Two-way ANOVA** | | | | | | | | | | | |
|  | | *Gene* | *Interaction, F* | *Interaction, p* | | *Genotype, F* | | *Genotype, p* | | *Sex, F* | *Sex, p* |
| mRNA | | Mbp | 0.50 | 0.49 | | 0.05 | | 0.83 | | 0.5 | 0.49 |
|  |  | Plp1 | 0.86 | 0.37 | | **26.91** | | **<0.01** | | 0.86 | 0.37 |
|  |  | Mag | 0.06 | 0.81 | | <0.01 | | >0.99 | | 0.06 | 0.81 |
|  |  | Mog | <0.01 | >0.99 | | 0.37 | | 0.55 | | <0.01 | >0.99 |
| Western blot | | Plp1 | 2.49 | 0.15 | | **331.2** | | **<0.01** | | 3.22 | 0.11 |
| **Tukey’s tests** | | | | | | | | | | | |
|  | *Gene* | | *WT ♂ vs ♀, p* | | *KO ♂ vs ♀, p* | | *♂ KO vs WT, p* | | *♀ KO vs WT, p* | | |
| mRNA | Mbp | | >0.99 | | 0.75 | | 0.91 | | 0.99 | | |
|  | Plp1 | | >0.99 | | 0.57 | | **<0.01** | | **<0.05** | | |
|  | Mag | | >0.99 | | 0.98 | | >0.99 | | >0.99 | | |
|  | Mog | | >0.99 | | >0.99 | | 0.97 | | 0.97 | | |
| Western blot | Plp1 | | >0.99 | | 0.16 | | **<0.01** | | **<0.01** | | |

***Table S4. Summary of comparisons in the measures of dominant and aggressive behavior after LPS-treatment (two-way ANOVA, post-hoc Tukey’s test. Chi-square and Fisher’s exact test, Fig. 2 A-F.***

| **Two-way ANOVA *(LPS groups, normalized to sex- and genotype-matched PBS groups)*** | | | | | | | | | | | | |
| --- | --- | --- | --- | --- | --- | --- | --- | --- | --- | --- | --- | --- |
| *Behavior* | *Parameter* | | *Interaction, F* | | *Interaction, p* | | *Genotype, F* | | | *Genotype, p* | *Sex, F* | *Sex, p* |
| ***Dominant and aggressive behavior*** | | | | | | | | | | | | |
| Following | Latency | | 1.01 | | 0.33 | | 1.86 | | | 0.19 | 0.92 | 0.35 |
|  | Duration | | **7.47** | | **0.01** | | 2.10 | | | 0.16 | 2.23 | 0.15 |
|  | Number | | **4.78** | | **0.04** | | 0.05 | | | 0.83 | 0.34 | 0.57 |
| Mounting | Latency | | 1.26 | | 0.30 | | 1.22 | | | 0.32 | 0.42 | 0.52 |
|  | Duration | | **6.39** | | **0.02** | | 0.76 | | | 0.39 | 0.76 | 0.39 |
|  | Number | | **4.77** | | **0.04** | | 0.66 | | | 0.43 | 1.41 | 0.25 |
| ***Neutral social*** ***exploration*** | | | | | | | | | | | | |
| N-A contact | Latency | | 0.13 | | 0.72 | | 0.50 | | | 0.49 | 0.05 | 0.83 |
|  | Duration | | 0.45 | | 0.51 | | 0.19 | | | 0.67 | <0.01 | 0.96 |
|  | Number | | 0.07 | | 0.79 | | 2.38 | | | 0.14 | 0.14 | 0.71 |
| N-N contact | Latency | | <0.01 | | 0.93 | | 1.44 | | | 0.24 | 0.93 | 0.35 |
|  | Duration | | 1.31 | | 0.27 | | 0.02 | | | 0.88 | 2.20 | 0.15 |
|  | Number | | 0.30 | | 0.59 | | <0.01 | | | 0.95 | 3.41 | 0.08 |
| **Tukey’s tests** | | | | | | | | | | | | |
| *Behavior* | | *Parameter* | | *WT ♂ vs ♀, p* | | *KO ♂ vs ♀, p* | | | *♂ KO vs WT, p* | | *♀ KO vs WT, p* | |
| ***Dominant and aggressive behavior*** | | | | | | | | | | | | |
| Following | | Latency | | 0.52 | | >0.99 | | | 0.36 | | >0.99 | |
|  |  | Duration | | **0.03** | | 0.82 | | | 0.80 | | **0.04** | |
|  |  | Number | | 0.24 | | 0.67 | | | 0.35 | | 0.52 | |
| Mounting | | Latency | | >0.99 | | >0.99 | | | >0.99 | | >0.99 | |
|  |  | Duration | | 0.11 | | 0.65 | | | 0.65 | | 0.11 | |
|  |  | Number | | 0.11 | | 0.89 | | | 0.77 | | 0.18 | |
| ***Neutral social exploration*** | | | | | | | | | | | | |
| N-A contact | | Latency | | 0.98 | | >0.99 | | | 0.87 | | >0.99 | |
|  |  | Duration | | 0.97 | | 0.96 | | | 0.86 | | >0.99 | |
|  |  | Number | | 0.97 | | >0.99 | | | 0.59 | | 0.80 | |
| N-N contact | | Latency | | 0.92 | | 0.88 | | | 0.80 | | 0.86 | |
|  |  | Duration | | 0.28 | | >0.99 | | | 0.89 | | 0.80 | |
|  |  | Number | | 0.35 | | 0.79 | | | 0.99 | | 0.97 | |
| **Chi square test** | | | | | | | | | | | | |
| *Parameter* | | *Males* | | | | | | *Females* | | | | |
| Incidence of following | | **Χ^2^(3, N=24)=13.33, p<0.01** | | | | | | Χ^2^(3, N=24)=4.444, p=0.22 | | | | |
| Incidence of mounting | | **Χ^2^(3, N=24)=22.3, p<0.01** | | | | | | **Χ^2^(3, N=24)=27.86, p<0.01** | | | | |
| Incidence of attacks | | **Χ^2^(3, N=24)=15.42, p<0.01** | | | | | | *Not measured* | | | | |

**Table S4 (continued).**

| **Fisher’s exact test** | | | | | | | | | | | | | | |
| --- | --- | --- | --- | --- | --- | --- | --- | --- | --- | --- | --- | --- | --- | --- |
| *Parameter* | *Males, p* | | | | | | | *Females, p* | | | | | | |
|  | WT  (PBS vs. LPS) | | KO  (PBS vs LPS) | | PBS  (WT vs. KO) | LPS  (WT vs. KO) | | WT  (PBS vs. LPS) | | KO  (PBS vs LPS) | | PBS  (WT vs. KO) | | LPS  (WT vs. KO) |
| Incidence of following | >0.99 | | **0.01** | | >0.99 | **0.01** | | 0.15 | | 0.47 | | >0.99 | | >0.99 |
| Incidence of mounting | >0.99 | | **0.01** | | >0.99 | **0.01** | | **<0.01** | | >0.99 | | >0.99 | | **<0.01** |
| Incidence of attacks | 0.61 | | **0.03** | | >0.99 | **<0.01** | | *Not measured* | | | | | | |
| **Unpaired t-test *(Male LPS groups, normalized to genotype-matched PBS groups)*** | | | | | | | | | | | | | | |
| Duration of attacks | | **t=2.230, df=10** | | | | | | | **p<0.05** | | | | | |
| **Two-way ANOVA *(Male LPS groups, normalized to WT PBS group)*** | | | | | | | | | | | | | | |
| *Parameter* | | *Interaction, F* | | *Interaction, p* | | | *Genotype, F* | | *Genotype, p* | | *Sex, F* | | *Sex, p* | |
| Number of attacks | | 4.19 | | >0.05 | | | 2.75 | | 0.11 | | 1.62 | | 0.22 | |
| Latency to attack | | 2.29 | | 0.15 | | | 1.94 | | 0.18 | | 1.61 | | 0.22 | |

***Table S5A*. *Summary of comparisons in the measures of cytokine expression in the brain and the spleen upon LPS administration normalized to sex-matched wild-type PBS controls (three-way ANOVA, Sidak’s test). Fig. 2 G-L.***

| **Three-way ANOVA** | | | | | | | | | | | | | | | | | | | | | | | | | | | | | | | | |
| --- | --- | --- | --- | --- | --- | --- | --- | --- | --- | --- | --- | --- | --- | --- | --- | --- | --- | --- | --- | --- | --- | --- | --- | --- | --- | --- | --- | --- | --- | --- | --- | --- |
| *Cytokine* | *LPS (L)* | | | *Sex (S)* | | | | *Genotype (G)* | | | | *L × S interaction* | | *L × G interaction* | | | | | | | *S × G interaction* | | | | | | | *L × S × G interaction* | | | | |
|  | *F* | *p* | | *F* | | *p* | | *F* | | *p* | | *F* | *p* | *F* | | | *p* | | | | *F* | | | *p* | | | | *F* | | | | *p* |
| ***Brain*** | | | | | | | | | | | | | | | | | | | | | | | | | | | | | | | | |
| IL-1β | **180.3** | **<0.01** | | **11.87** | | **<0.01** | | <0.01 | | 0.93 | | **11.33** | **<0.01** | **10.50** | | | **<0.01** | | | | **55.98** | | | **<0.01** | | | | **57.18** | | | | **<0.01** |
| IL-6 | **212.8** | **<0.01** | | **21.97** | | **<0.01** | | **109.5** | | **<0.01** | | **30.25** | **<0.01** | **132.0** | | | **<0.01** | | | | **13.14** | | | **<0.01** | | | | **7.91** | | | | **<0.01** |
| TNF | **815.2** | **<0.01** | | **500.5** | | **<0.01** | | **280.4** | | **<0.01** | | **331.0** | **<0.01** | **427.9** | | | **<0.01** | | | | **166.1** | | | **<0.01** | | | | **291.2** | | | | **<0.01** |
| ***Spleen*** | | | | | | | | | | | | | | | | | | | | | | | | | | | | | | | | |
| IL-1β | **80.45** | **<0.01** | | 1.57 | | 0.22 | | **5.28** | | **0.03** | | **4.84** | **0.04** | 0.25 | | | 0.62 | | | **27.40** | | | | **<0.01** | | | | **18.39** | | | | **<0.01** |
| IL-6^†^ | **263.8** | **<0.01** | | 1.54 | | 0.23 | | **57.41** | | **<0.01** | | 1.97 | 0.17 | **51.43** | | | **<0.01** | | | 0.14 | | | | 0.71 | | | | 0.30 | | | | 0.59 |
| TNF | **84.08** | **<0.01** | | **18.41** | | **<0.01** | | **34.39** | | **<0.01** | | **8.67** | **<0.01** | **59.13** | | | **<0.01** | | | **31.93** | | | | **<0.01** | | | | **18.53** | | | | **<0.01** |
| **Sidak’s tests** | | | | | | | | | | | | | | | | | | | | | | | | | | | | | | | | |
| *Cytokine* | Males vs. females | | | | | | | | Wild type vs. KO | | | | | | | | | PBS vs. LPS | | | | | | | | | | | | | | |
|  | *WT PBS*  *(♂ vs ♀)* | | *KO PBS*  *(♂ vs ♀)* | | WT LPS  *(♂ vs ♀)* | | KO LPS  *(♂ vs ♀)* | | *♂ PBS*  (WT vs KO) | | *♂ LPS*  (WT vs KO) | | *♀ PBS*  *(WT vs. KO)* | | *♀ LPS*  *(WT vs. KO)* | | | *♂ WT*  *(PBS vs. LPS)* | | | | *♂ KO*  *(PBS vs. LPS)* | | | *♀ WT*  *(PBS vs. LPS)* | | | | *♀ KO*  *(PBS vs. LPS)* | | | |
| ***Brain*** | | | | | | | | | | | | | | | | | | | | | | | | | | | | | | | | |
| IL-1β | >0.99 | | >0.99 | | **<0.01** | | **<0.01** | | 0.79 | | **<0.01** | | 0.75 | | | **<0.01** | | | **<0.01** | | | | 0.07 | | | | **0.03** | | | | **<0.01** | |
| IL-6 | >0.99 | | >0.99 | | **<0.01** | | 0.57 | | >0.99 | | **<0.01** | | >0.99 | | | **<0.01** | | | **<0.01** | | | | 0.08 | | | | **<0.01** | | | | >0.99 | |
| TNF | >0.99 | | **<0.01** | | **<0.01** | | **<0.01** | | **<0.01** | | **<0.01** | | >0.99 | | | **<0.01** | | | **<0.01** | | | | **<0.01** | | | | **<0.01** | | | | **0.01** | |
| ***Spleen*** | | | | | | | | | | | | | | | | | | | | | | | | | | | | | | | | |
| IL-1β | >0.99 | | >0.99 | | **<0.01** | | >0.05 | | >0.99 | | **<0.01** | | 0.90 | | | **<0.01** | | | **<0.01** | | | | **<0.01** | | | 0.98 | | | | **<0.01** | | |
| IL-6 | >0.99 | | >0.99 | | >0.99 | | 0.59 | | >0.99 | | **<0.01** | | >0.99 | | | **<0.01** | | | **<0.01** | | | | **<0.01** | | | **<0.01** | | | | **<0.01** | | |
| TNF | >0.99 | | 0.96 | | 0.87 | | **<0.01** | | 0.80 | | 0.49 | | >0.99 | | | **<0.01** | | | 0.80 | | | | **<0.01** | | | >0.99 | | | | **<0.01** | | |

† see **Table S5B** for two-way ANOVA.

***Table S5B*. *Summary of comparisons of consolidated data on expression of IL-6 in the spleen upon LPS administration normalized to sex-matched wild-type PBS controls (two-way ANOVA, Tukey’s test). Fig. 2 G-L.***

| *IL-6 expression in the spleen* | | | |
| --- | --- | --- | --- |
| **Two-way ANOVA (treatment × genotype interaction)** | | | |
| *Interaction, F* | | *Interaction, p* | |
| **55.73** | | **<0.01** | |
| **Tukey’s test** | | | |
| PBS WT vs. KO | LPS WT vs. KO | WT PBS vs. LPS | KO PBS vs. LPS |
| >0.99 | **<0.01** | **<0.01** | **<0.01** |

***Table S6A.* *Summary of comparison in the measures of dominant and aggressive behavior after LPS treatment normalized to a sex-matched wild-type PBS control group (three-way ANOVA, Sidak’s test). Fig. S6-S7.***

| **Three-way ANOVA** | | | | | | | | | | | | | | | | | | | | | | | | | | | | | | | | | | | | | | | | | | | | | | |
| --- | --- | --- | --- | --- | --- | --- | --- | --- | --- | --- | --- | --- | --- | --- | --- | --- | --- | --- | --- | --- | --- | --- | --- | --- | --- | --- | --- | --- | --- | --- | --- | --- | --- | --- | --- | --- | --- | --- | --- | --- | --- | --- | --- | --- | --- | --- |
| *Behavior* | *Parameter* | *LPS (L)* | | | | | *Sex (S)* | | | | | | | *Genotype (G)* | | | | | | | | *L × S interaction* | | | | | | *L × G interaction* | | | | | | | *S × G interaction* | | | | | | | *L × S × G interaction* | | | | |
|  |  | *F* | *p* | | | | *F* | | | *p* | | | | *F* | | | *p* | | | | | *F* | | | | *p* | | *F* | | | *p* | | | | *F* | | | *p* | | | | *F* | | | | *p* |
| ***Dominant and aggressive behavior*** | | | | | | | | | | | | | | | | | | | | | | | | | | | | | | | | | | | | | | | | | | | | | | |
| Following | Latency | 2.79 | 0.09 | | | | 1.10 | | | 0.30 | | | | 1.75 | | | 0.19 | | | | | 0.23 | | | | 0.63 | | 0.59 | | | 0.45 | | | | 0.09 | | | 0.76 | | | | 0.76 | | | | 0.39 |
|  | Duration^†^ | **8.49** | **<0.01** | | | | **11.65** | | | **<0.01** | | | | 0.72 | | | 0.40 | | | | | **4.39** | | | | **0.04** | | 0.15 | | | 0.70 | | | | 0.12 | | | 0.73 | | | | 0.95 | | | | 0.34 |
|  | Number | **5.57** | **0.02** | | | | **5.57** | | | **0.02** | | | | **5.50** | | | **0.02** | | | | | 1.20 | | | | 0.28 | | 1.04 | | | 0.32 | | | | 0.81 | | | 0.37 | | | | 0.14 | | | | 0.71 |
| Mounting | Latency^†^ | 3.56 | 0.07 | | | | 0.57 | | | 0.45 | | | | 1.97 | | | 0.17 | | | | | 0.49 | | | | 0.49 | | <0.01 | | | 0.96 | | | | **6.70** | | | **0.01** | | | | 1.78 | | | | 0.26 |
|  | Duration | 3.33 | 0.08 | | | | 0.60 | | | 0.44 | | | | 0.30 | | | 0.35 | | | | | 0.90 | | | | 0.35 | | 0.60 | | | 0.44 | | | | **6.72** | | | **0.01** | | | | **5.83** | | | | **0.02** |
|  | Number | 3.35 | 0.07 | | | | 0.90 | | | 0.35 | | | | 1.15 | | | 0.29 | | | | | 1.44 | | | | 0.24 | | 0.67 | | | 0.42 | | | | **6.05** | | | **0.02** | | | | **4.87** | | | | **0.03** |
| ***Neutral social exploration*** | | | | | | | | | | | | | | | | | | | | | | | | | | | | | | | | | | | | | | | | | | | | | | |
| N-A contact | Latency | <0.01 | 0.93 | | | 1.27 | | | | 0.27 | | | 0.07 | | | | 0.79 | | | | <0.01 | | | | 0.98 | | 0.17 | | | | 0.67 | | | | 2.20 | | | 0.15 | | | | 0.14 | | | | 0.71 |
|  | Duration | 2.71 | 0.11 | | | 1.09 | | | | 0.30 | | | **4.52** | | | | **0.04** | | | | 0.03 | | | | 0.87 | | 0.56 | | | | 0.46 | | | | 0.46 | | | 0.50 | | | | 0.04 | | | | 0.84 |
|  | Number | 3.19 | 0.08 | | | 0.48 | | | | 0.49 | | | **4.09** | | | | **<0.05** | | | | 0.17 | | | | 0.68 | | 2.13 | | | | 0.15 | | | | 0.03 | | | 0.86 | | | | 0.01 | | | | 0.92 |
| N-N contact | Latency | <0.01 | 0.96 | | | 0.35 | | | | 0.56 | | | 0.45 | | | | 0.51 | | | | 0.61 | | | | 0.44 | | 0.77 | | | | 0.38 | | | | 1.03 | | | 0.32 | | | | 0.13 | | | | 0.72 |
|  | Duration | 0.18 | 0.67 | | | 0.93 | | | | 0.34 | | | 0.04 | | | | 0.83 | | | | 1.35 | | | | 0.25 | | 0.02 | | | | 0.90 | | | | 1.22 | | | 0.28 | | | | 0.82 | | | | 0.37 |
|  | Number | 0.32 | 0.58 | | | 0.33 | | | | 0.57 | | | 1.62 | | | | 0.21 | | | | 2.03 | | | | 0.16 | | <0.01 | | | | 0.96 | | | | 1.72 | | | 0.20 | | | | 0.22 | | | | 0.64 |
| **Sidak’s tests** | | | | | | | | | | | | | | | | | | | | | | | | | | | | | | | | | | | | | | | | | | | | | | |
|  |  | Males vs. females | | | | | | | | | | | | | | Wild type vs. KO | | | | | | | | | | | | | | | | | | PBS vs. LPS | | | | | | | | | | | | |
| *Behavior* | *Parameter* | *WT PBS*  *(♂ vs ♀)* | | *KO PBS*  *(♂ vs ♀)* | | | | WT LPS  *(♂ vs ♀)* | | | KO LPS  *(♂ vs ♀)* | | | | *♂ PBS*  (WT vs KO) | | | *♂ LPS*  (WT vs KO) | | | | | *♀ PBS*  *(WT vs. KO)* | | | | | | *♀ LPS*  *(WT vs. KO)* | | | *♂ WT*  *(PBS vs. LPS)* | | | | *♂ KO*  *(PBS vs. LPS)* | | | *♀ WT*  *(PBS vs. LPS)* | | | | *♀ KO*  *(PBS vs. LPS)* | | | |
| ***Dominant and aggressive behavior*** | | | | | | | | | | | | | | | | | | | | | | | | | | | | | | | | | | | | | | | | | | | | | | |
| **Following** | Latency | >0.99 | | | >0.99 | | | | 0,91 | | | >0.99 | | | | >0.99 | | | 0.76 | | | | | >0.99 | | | | | | >0.99 | | | >0.99 | | | | 0.87 | | | | 0.97 | | | | 0.99 | |
|  | Duration | >0.99 | | | 0.93 | | | | **<0.05** | | | 0.21 | | | | >0.99 | | | >0.99 | | | | | 0.94 | | | | | | >0.99 | | | >0.99 | | | | >0.99 | | | | **0.03** | | | | 0.61 | |
|  | Number | >0.99 | | | 0.94 | | | | 0.86 | | | 0.48 | | | | >0.99 | | | 0.88 | | | | | 0.93 | | | | | | 0.52 | | | >0.99 | | | | 0.90 | | | | 0.62 | | | | 0.40 | |
| **Mounting** | Latency | >0.99 | | | 0.86 | | | | 0.60 | | | 0.56 | | | | >0.99 | | | 0.96 | | | | | 0.86 | | | | | | 0.17 | | | >0.99 | | | | 0.96 | | | | 0.60 | | | | >0.99 | |
|  | Duration | >0.99 | | | >0.99 | | | | **0.02** | | | 0.75 | | | | >0.99 | | | 0.75 | | | | | >0.99 | | | | | | **0.02** | | | >0.99 | | | | 0.95 | | | | 0.06 | | | | >0.99 | |
|  | Number | >0.99 | | | >0.99 | | | | **0.02** | | | 0.94 | | | | >0.99 | | | 0.90 | | | | | >0.99 | | | | | | **0.03** | | | >0.99 | | | | >0.99 | | | | >0.05 | | | | >0.99 | |
| ***Neutral social exploration*** | | | | | | | | | | | | | | | | | | | | | | | | | | | | | | | | | | | | | | | | | | | | | | |
| **N-A contacts** | Latency | >0.99 | | | 0.98 | | | | >0.99 | | | 0.84 | | | | >0.99 | | | | 0.94 | | | | | >0.99 | | | | | >0.99 | | | >0.99 | | | | >0.99 | | | >0.99 | | | | >0.99 | | |
|  | Duration | >0.99 | | | >0.99 | | | | >0.99 | | | >0.99 | | | | >0.99 | | | | 0.96 | | | | | 0.98 | | | | | 0.73 | | | >0.99 | | | | 0.96 | | | >0.99 | | | | 0.97 | | |
|  | Number | >0.99 | | | >0.99 | | | | >0.99 | | | >0.99 | | | | >0.99 | | | | 0.7 | | | | | >0.99 | | | | | 0.65 | | | >0.99 | | | | 0.86 | | | >0.99 | | | | 0.65 | | |
| **N-N contacts** | Latency | >0.99 | | | 0.9 | | | | >0.99 | | | >0.99 | | | | 0.86 | | | | >0.99 | | | | | >0.99 | | | | | >0.99 | | | >0.99 | | | | >0.99 | | | >0.99 | | | | >0.99 | | |
|  | Duration | >0.99 | | | >0.99 | | | | 0.42 | | | >0.99 | | | | >0.99 | | | | >0.99 | | | | | >0.99 | | | | | 0.97 | | | >0.99 | | | | >0.99 | | | 0.93 | | | | >0.99 | | |
|  | Number | >0.99 | | | >0.99 | | | | 0.56 | | | >0.99 | | | | >0.99 | | | | >0.99 | | | | | 0.99 | | | | | 0.81 | | | 0.96 | | | | >0.99 | | | >0.99 | | | | >0.99 | | |

† see **Table S6B** for two-way ANOVA.

***Table S6B*. *Summary of comparisons of consolidated data on behavior and cytokine expression after LPS treatment normalized to a sex-matched wild-type PBS control group (two-way ANOVA, Tukey’s test). Fig. S6-S7.***

| ***Dominant and aggressive behavior*** | | | | | | |
| --- | --- | --- | --- | --- | --- | --- |
| *Behavior* | *Interaction* | | *Interaction, F* | | *Interaction, p* | |
| **Two-way ANOVA** | | | | | | |
| Following duration | Treatment × Sex | | **4.61** | | **0.04** | |
| Latency to mounting | Sex × Genotype | | **6.50** | | **0.01** | |
| **Tukey’s test** | | | | | | |
| Following duration | PBS *♂* vs. *♀* | LPS *♂* vs. *♀* | | PBS *♂* vs. *♂* | | PBS *♀* vs. *♀* |
|  | 0.78 | **<0.01** | | 0.93 | | **<0.01** |
| Latency to mounting | *♂* WT vs. KO | *♀* WT vs. KO | | WT *♂* vs. *♀* | | KO *♂* vs. *♀* |
|  | 0.84 | **0.04** | | 0.58 | | 0.11 |

**Table S7. Summary of comparisons of the measures of cytokine expression in the brain and the spleen upon LPS administration normalized to sex- and genotype-matched PBS control groups (two-way ANOVA, Tukey’s test). Fig S8.**

| **Two-way ANOVA** | | | | | | | | |
| --- | --- | --- | --- | --- | --- | --- | --- | --- |
| *Gene* | *Interaction, F* | *Interaction, p* | | *Genotype, F* | *Genotype, p* | *Sex, F* | | *Sex, p* |
| ***Brain*** | | | | | | | | |
| IL-1β | **129.8** | **<0.01** | | **250.1** | **<0.01** | **112.0** | | **<0.01** |
| IL-6 | **15.09** | **<0.01** | | **246.6** | **<0.01** | **53.70** | | **<0.01** |
| TNF | **587.6** | **<0.01** | | **808.6** | **<0.01** | **435.7** | | **<0.01** |
| ***Spleen*** | | | | | | | | |
| IL-1β | **16.07** | **<0.01** | | **15.40** | **<0.01** | **21.73** | | **<0.01** |
| IL-6 | **4.951** | **0.04** | | **13.39** | **<0.01** | **11.86** | | **<0.01** |
| TNF^†^ | 3.507 | 0.08 | | **105.3** | **<0.01** | 0.37 | | 0.55 |
| **Tukey’s test** | | | | | | | | |
| *Gene* | *WT ♂ vs ♀, p* | | *KO ♂ vs ♀, p* | | *♂ KO vs WT, p* | | *♀ KO vs WT, p* | |
| ***Brain*** | | | | | | | | |
| IL-1β | **<0.01** | | 0.94 | | **<0.01** | | **0.02** | |
| IL-6 | **<0.01** | | 0.11 | | **<0.01** | | **<0.01** | |
| TNF | **<0.01** | | 0.09 | | **<0.01** | | **0.03** | |
| ***Spleen*** | | | | | | | | |
| IL-1β | **<0.01** | | 0.97 | | **<0.01** | | >0.99 | |
| IL-6 | **<0.01** | | 0.82 | | 0.74 | | **<0.01** | |
| TNF | 0.79 | | 0.35 | | **<0.01** | | **<0.01** | |

**Table S8. Comparison of blood count of mononuclear (MNC) and polymorphonuclear (PMNC) white blood cells in male and female WT vs. ST3-deficient mice (x10^4^/ml)^1^**

| **Genotype** | **Sex** | **MNC** | **PMNC** |
| --- | --- | --- | --- |
| WT | Male | **178 ± 53** | **47 ± 20** |
| ST3^-/-^ | Male | **201 ± 66** | **51 ± 18** |
| WT | Female | **220 ± 63** | **55 ± 17** |
| ST3^-/-^ | Female | **190 ± 70** | **45 ± 15** |

^1^Blood was collected via cardiac puncture using 0.38% od sodium citrate as anticoagulant, red blood cells were removed using ACK erythrocyte lysis media (ThermoFisher Scientific – Hong Kong, Hong Kong), washed in PBS and MNC and PMNC were separated using Ficoll^TM^ Plaque Plus (Sigma, St. Louis, MO) gradient according to manufacturer’ recommendation and both cell types were reconstituted in the volume of PBS equal to initial blood volume. The cells were counted using haemocytometer, mean ± SE is shown (n=3-4).

**References**

ARRIVE guidelines [WWW Document], n.d. URL https://arriveguidelines.org/ (accessed 6.21.21).

Couch, Y., Trofimov, A., Markova, N., Nikolenko, V., Steinbusch, H.W., Chekhonin, V., Schroeter, C., Lesch, K.P., Anthony, D.C., Strekalova, T., 2016. Low-dose lipopolysaccharide (LPS) inhibits aggressive and augments depressive behaviours in a chronic mild stress model in mice. J. Neuroinflammation 13, 1–17. https://doi.org/10.1186/s12974-016-0572-0

de Munter, J., Schafarevich, I., Liundup, A., Pavlov, D., Wolters, E., Gorlova, A., Veniaminova, E., Umriukhin, A., Kalueff, A., Svistunov, A., Kramer, B.W., Lesch, K.-P., Strekalova, T., 2020. Neuro-Cell therapy improves motor outcomes and suppresses inflammation during experimental syndrome of amyotrophic lateral sclerosis in mice. CNS Neurosci. Ther.

Dukhinova, M., Kuznetsova, I., Kopeikina, E., Veniaminova, E., Yung, A.W.Y., Veremeyko, T., Levchuk, K., Barteneva, N.S., Wing-Ho, K.K., Yung, W.H., Liu, J.Y.H., Rudd, J., Yau, S.S.Y., Anthony, D.C., Strekalova, T., Ponomarev, E.D., 2018. Platelets mediate protective neuroinflammation and promote neuronal plasticity at the site of neuronal injury. Brain. Behav. Immun. 74, 7–27. https://doi.org/10.1016/j.bbi.2018.09.009

Dukhinova, M., Veremeyko, T., Yung, A.W.Y., Kuznetsova, I.S., Lau, T.Y.B., Kopeikina, E., Chan, A.M.L., Ponomarev, E.D., 2019. Fresh evidence for major brain gangliosides as a target for the treatment of Alzheimer’s disease. Neurobiol. Aging 77, 128–143. https://doi.org/10.1016/j.neurobiolaging.2019.01.020

Gorlova, A., Ortega, G., Waider, J., Bazhenova, N., Veniaminova, E., Proshin, A., Kalueff, A. V., Anthony, D.C., Lesch, K.P., Strekalova, T., 2020. Stress-induced aggression in heterozygous TPH2 mutant mice is associated with alterations in serotonin turnover and expression of 5-HT6 and AMPA subunit 2A receptors. J. Affect. Disord. 272, 440–451. https://doi.org/10.1016/j.jad.2020.04.014

Gorlova, A., Pavlov, D., Anthony, D.C., Ponomarev, E.D., Sambon, M., Proshin, A., Shafarevich, I., Babaevskaya, D., Lesсh, K.P., Bettendorff, L., Strekalova, T., 2019. Thiamine and benfotiamine counteract ultrasound-induced aggression, normalize AMPA receptor expression and plasticity markers, and reduce oxidative stress in mice. Neuropharmacology 156, 107543. https://doi.org/10.1016/j.neuropharm.2019.02.025

Kopeikina, E., Dukhinova, M., Yung, A.W.Y., Veremeyko, T., Kuznetsova, I.S., Lau, T.Y.B., Levchuk, K., Ponomarev, E.D., 2020. Platelets promote epileptic seizures by modulating brain serotonin level, enhancing neuronal electric activity, and contributing to neuroinflammation and oxidative stress. Prog. Neurobiol. 188, 101783. https://doi.org/10.1016/j.pneurobio.2020.101783

Lim, L.W., Shrestha, S., Or, Y.Z., Tan, S.Z.K., Chung, H.H., Sun, Y., Lim, C.L., Khairuddin, S., Lufkin, T., Lin, V.C.L., 2016. Tetratricopeptide repeat domain 9A modulates anxiety-like behavior in female mice. Sci. Rep. 6, 37568. https://doi.org/10.1038/srep37568

Sotnikov, I., Veremeyko, T., Starossom, S.C., Barteneva, N., Weiner, H.L., Ponomarev, E.D., 2013. Platelets Recognize Brain-Specific Glycolipid Structures, Respond to Neurovascular Damage and Promote Neuroinflammation. PLoS One 8. https://doi.org/10.1371/journal.pone.0058979

Strekalova, T., 2008. Optimization of the chronic stress depression model in C57 BL/6 mice: evidences for improved validity, in: J, L. (Ed.), Behavioral Models in Stress Research. Volume I. Nova Science Publishers, NY, USA, pp. 95–139.

Strekalova, T., Steinbusch, H., 2009. Factors of Reproducibility of Anhedonia Induction in a Chronic Stress Depression Model in Mice, in: Gould, T.D. (Ed.), Mood and Anxiety Related Phenotypes in Mice: Characterization Using Behavioral Tests, Neuromethods. Humana Press, Totowa, NJ, pp. 153–176. https://doi.org/10.1007/978-1-60761-303-9_9

Veniaminova, E., Cespuglio, R., Chernukha, I., Schmitt-Boehrer, A.G., Morozov, S., Kalueff, A. V., Kuznetsova, O., Anthony, D.C., Lesch, K.-P., Strekalova, T., 2020. Metabolic, Molecular, and Behavioral Effects of Western Diet in Serotonin Transporter-Deficient Mice: Rescue by Heterozygosity? Front. Neurosci. 14. https://doi.org/10.3389/fnins.2020.00024

Veniaminova, E., Cespuglio, R., Cheung, C.W., Umriukhin, A., Markova, N., Shevtsova, E., Lesch, K.P., Anthony, D.C., Strekalova, T., 2017. Autism-Like Behaviours and Memory Deficits Result from a Western Diet in Mice. Neural Plast. 2017. https://doi.org/10.1155/2017/9498247

Veremeyko, T., Yung, A.W.Y., Anthony, D.C., Strekalova, T., Ponomarev, E.D., 2018. Early Growth Response Gene-2 Is Essential for M1 and M2 Macrophage Activation and Plasticity by Modulation of the Transcription Factor CEBPβ. Front. Immunol. 9. https://doi.org/10.3389/fimmu.2018.02515
